# Nuclear gasdermin E drives endothelin-1-induced metastatic progression independently of the pyroptosis

**DOI:** 10.1038/s41419-025-08202-x

**Published:** 2026-01-16

**Authors:** Celia Roman, Valentina Caprara, Piera Tocci, Andrea Sacconi, Giovanni Blandino, Anna Bagnato, Rosanna Sestito

**Affiliations:** 1 Preclinical Models and New Therapeutic Agents Unit, Istituto di Ricovero e Cura a Carattere Scientifico (IRCCS), Regina Elena National Cancer Institute, Rome, Italy; 2 Translational Oncology Research Unit, IRCCS, Regina Elena National Cancer Institute, Rome, Italy

**Keywords:** Ovarian cancer, Mechanisms of disease

## Abstract

Elucidation of the molecular mechanism underlying metastatic dissemination in patients with high-grade serous ovarian carcinoma (HG-SOC) has the potential to affect patient outcome. This study explores the role of gasdermins (GSDMs) in HG-SOC, focusing on novel pyroptosis-independent nuclear functions of GSDME, which are integrated with the endothelin-1 (ET-1)/ET-1 receptor A (ET_A_R) signaling to sustain metastatic progression. In this tumor, GSDME upregulation is correlated to epithelial-mesenchymal transition (EMT) and ET_A_R expression. ET-1 signaling fuels GSDME expression by inducing its transcription via the core EMT factors, ZEB1 and ZEB2. GSDME, in turn, translocates to the nucleus to engage ZEB1 and transcriptionally regulate genes coupled with EMT and inflammatory signals, such as E-cadherin, vimentin and interleukin (IL)-6. GSDME depletion, similarly to ZEB1 and ET_A_R blockade, restrains ET-1-induced EMT phenotypic plasticity and inflammatory cytokine release. Clinically relevant, ET-1 receptor (ET-1R) antagonist, by depleting the nuclear reservoir of the GSDME/ZEB1 transcriptional complex, hinders the metastatic traits of HG-SOC. The intertwined ET_A_R/GSDME/ZEB1 circuitry characterizes mesenchymal HG-SOC patients and associates with a high-risk of poor survival. Together, these findings unveil GSDME as a key transcriptional regulator of aggressive behaviors and worse prognosis in HG-SOC patients, in an ET-1-driven alliance with ZEB1, which could be targeted by ET-1R antagonist to reduce the metastatic burden of this tumor.

## Introduction

High-grade serous ovarian carcinoma (HG-SOC) is a highly aggressive and deadly subtype of epithelial ovarian cancer, primarily due to its aggressive nature and late-stage diagnosis [1–3]. Even with progress in treatments and surgical procedures, the chances of long-term survival for HG-SOC patients remain poor, as the disease often recurs. Current initial treatments for HG-SOC involve surgical tumor debulking followed by platinum-based regimens [3, 4]. However, the effectiveness of these treatments is often limited when metastases refractory to therapy occur, as these therapeutic approaches are still ineffective in preventing HG-SOC metastases. Therefore, the characterization of the molecular events underlying the development of metastasis in HG-SOC has the potential to provide novel therapeutic strategies that impact patient outcome.

Advanced HG-SOC is characterized by the aberrant activation of the epithelial-to-mesenchymal transition (EMT) program and elevated levels of EMT genes are linked to reduced survival of HG-SOC patients [5]. EMT plasticity is controlled by an intricate landscape of regulatory circuits involving co-factors and signaling molecules that rely on transcription factor (TF) activity [6–9] to confer the aggressive nature of ovarian cancer.

Among the factors regulating EMT, the endothelin-1 (ET-1) signaling, consisting of ET-1 and its G protein-coupled receptor type A (ET_A_R) and B (ET_B_R), is emerging [10–13]. The master EMT-TF zinc finger E-box bind homeobox 1 (ZEB1) is prominently involved in generating ET-1/ET_A_R axis-driven transcriptional outputs that sustain tumor progression in HG-SOC [10, 14, 15].

Recently, it has been advised that some chemotherapeutic agents may lead to pyroptosis in certain tumor contexts, including ovarian cancer [16–21]. Pyroptosis is an inflammatory cell death that is triggered by gasdermins (GSDMs) [20]. GSDM family includes GSDMA-E and Pejvakin (PJVK), which mainly own the N-terminal pore-forming and the C-terminal inhibitory domains. Under pyroptosis-induced conditions, GSDMs are cleaved and the N-terminal fragment is released to allow the discharge of inflammatory cytokines and ultimately disruption of cell membrane integrity [20]. However, recent evidence has uncovered pyroptosis-independent roles of GSDMs in tumorigenesis [22–24], highlighting the complexity of GSDMs in cancer. Among GSDMs, ubiquitous expression of GSDME has been reported in ovarian cancer [16, 25–29]. Nowadays, the function of GSDME in metastatic progression has been poorly defined and an in-deep knowledge of GSDME role may thus provide critical insights for the development of effective therapies for metastatic HG-SOC.

Herein, we provide novel concepts on the acquisition of metastatic competence in HG-SOC, focusing on the unexplored nuclear activity of GSDME, driven by ET-1/ET_A_R axis, to generate ZEB1-related transcriptional programs and promote the acquisition of aggressive traits. At the mechanistic level, GSDME acts as a transcriptional co-regulator of ZEB1 affecting genes involved in EMT and inflammation, including E-cadherin, vimentin and interleukin (IL)-6. Targeting GSDME using an ET-1 receptor (ET-1R) antagonist offers a therapeutic strategy to hinder metastatic progression in HG-SOC.

## Materials and Methods

### Cell culture and reagents

In this study, we used primary cultures of patient-derived HG-SOC cells (PD HG-SOC), PMOV10, which were previously isolated from the ascitic fluid and characterized [30]. Moreover, we employed the following human HG-SOC cell lines: OVCA433 cells, which were obtained by Prof. G. Scambia (Catholic University School of Medicine, Rome, Italy), CAOV3 and Kuramochi cells, which were purchased from American Type Culture Collection (ATCC, Manassas, VA, USA #HTB-75), and Japanese Collection of Research Bioresources Cell Bank (JCRB, Osaka, Japan, #JCRB0098), respectively. Furthermore, we utilized a human breast cancer cell line, MDA-MB-231 (#HTB-26), and a human colon cancer cell line, SW620 (#CCL-227), purchased from ATCC. PD HG-SOC, Kuramochi and SW620 cells were cultured in RPMI-1640 medium (Thermo Fisher Scientific, Waltham, MA, USA, #618700010), whereas OVCA433, CAOV3, and MDA-MB-231 cells were cultured in DMEM (Thermo Fisher Scientific, #21885025). 10% fetal bovine serum (Thermo Fisher Scientific, #10270-106) and 1% penicillin-streptomycin (Euroclone, Pero, Italy, #ECB3001D) were added to the media, and cells were grown under a humidified atmosphere of 5% CO_2_ at 37 °C. Cells were authenticated through the short tandem repeat (STR) profiling, performed by IRCCS Ospedale Policlinico San Martino of Genova (Italy), and tested for mycoplasma infection, through the MycoGenie kit (Assay Genie, Dublin, Ireland, #MORV0011-50). Before each experiment, cells were incubated in serum-free medium for 24 h. ET-1 was used at 100 nM and was acquired by Sigma-Aldrich (Steinheim, Germany, #E7764). Macitentan (Selleck Chemicals, Houston, TX, USA, #S8051), BQ123 (Bachem, Bubendorf, Switzerland, #50-259-479), and BQ788 (MedChemExpress, Monmouth Junction, NJ, USA, #HY-15894A) were used at a dosage of 1 μM, 30 min before the stimulation with ET-1.

### RNA interference and plasmid transfection

Cells were transfected with Dharmacon smartpool siRNAs against ZEB1 (si-ZEB1, #L-006564-01) or GSDME (si-GSDME, #L-011844-00), or with control siRNAs (si-Ctr, #D-001810-10) (GE Healthcare Life Sciences, Marlborough, MA, USA) used at 100 nM concentration for 48/72 h. Lipofectamine RNAiMAX transfectant (Thermo Fisher Scientific, #13778150) was employed following the procedure suggested by the manufacturer. In parallel, cells were 24 h transfected by using 1-5 μg of flag-tagged ZEB1 (hZEB1), kindly provided by D. Dean [31], myc-tagged SIP1 (hZEB2), a kind gift from Dr D. Huylebroeck [32], flag-tagged SNAIL (hSNAIL, Addgene, Cambridge, MA, USA, #16218), or myc-flag-tagged DFNA5 (hGSDME, Origene, #RC225854) expression vectors. pCMV6 vector was utilized as control (Mock) and Lipofectamine 2000 (Thermo Fisher Scientific, #11668019) as transfecting reagent, following the instructions of the manufacturer.

### Western blot and immunoprecipitation analyses

To obtain cytoplasmic and nuclear cell lysates, a cell fractionation kit (Cell Signaling Technology, Danvers, MA, USA, #BK9038) was used, following the instructions of the manufacturer. Whole cell lysates were extracted in RIPA buffer (Cell Signaling Technology, #9806) complemented with protease (#11697498001) and phosphatase (#4906845001) inhibitors (Roche, Basel, Switzerland). Bio-Rad Protein Assay kit (Bio-Rad, Hercules, CA, USA, #5000001) was employed for protein quantification. Anti-histone H3 (D1H2, Cell Signaling Technology, #4499) and anti-α-tubulin (DM1A, Santa Cruz Biotechnology, Dallas, TX, USA, #sc-32293) antibodies (Abs) were employed to control the loading of nuclear and cytoplasmic lysates, respectively. Anti-β-actin (AC-15, Sigma Aldrich, #1978) Ab was employed to normalize total cell fractions. Immunoblotting (IB) analyses were performed as previously indicated [15] and by using the pre-casted 4–20% SDS/PAGE gels (Bio-Rad, #4561093) and the Trans-Blot transfer pack (Bio-Rad, #1704158). Co-immunoprecipitation (co-IP) assays were carried out as previously reported [15] and by using 150 μg of nuclear lysates or 500 μg of whole lysates and 1μg of anti-ZEB1 (H3, Santa Cruz Biotechnology, #sc-515797) or anti-mouse IgG Isotype Control (Thermo Fisher Scientific, #31903) Abs. Since IB signals of GSDME appeared near the heavy chain of IgG, HRP-conjugated protein A peroxidase (Pierce, Thermo Fisher Scientific, #32490) rather than HRP-conjugated secondary Ab was used. Blots were developed with the Clarity Western Blot ECL substrates (Bio-Rad, #1705061-2) and with the ChemiDoc Image System (BioRad). All the Abs used in IB and IP assays are listed in Supplementary Information, Table S1.

### RNA isolation and quantitative real-time PCR analysis

Trizol reagent (Thermo Fisher Scientific, #15596026) was employed for total RNA extraction following the procedure indicated by the manufacturer. The inspection of RNA purity and integrity, its quantization and retro-transcription were conducted as previously reported [15]. The mRNA expression of GSDME, E-cadherin, vimentin, IL-6, and cyclophilin-A was evaluated by quantitative real-time PCR (qRT-PCR) using the Luna Universal master mix (New England Biolabs, Ipswich, MA, USA, #M3003S) and the QuantStudio 6-Flex Real-Time PCR System (Thermo Fisher Scientific). Gene expression was determined through the ΔΔCt method, with cyclophilin-A expression serving as normalizer. Primer sequences are listed in Supplementary Information, Table S2.

### Luciferase reporter gene assay

PD HG-SOC cells (6 × 10^4^) were seeded in plates of 12 wells and were transfected with luciferase reporter vectors (500 ng) and Lipofectamine 2000. The luciferase activity of GSDME and IL-6 promoters was analyzed by employing constructs including a 1500 bp sequence from GSDME promoter (-1489/+11) or a 1350 bp sequence from IL-6 promoter (-1450/-101), synthetized by GenePharma (Shangai, China) and NeoBiotech (Nanterre, France), respectively. To measure the E-cadherin promoter activity a pGL2-EcadK1 plasmid (-108/+125 E-cadherin promoter sequence), given by Dr. E.R. Fearon (University of Michigan, USA), was used. pCMV-β-galactosidase vector (250 ng; Promega) was co-transfected with the reported constructs in the presence or not of siRNAs or expression plasmids. After 24 h of transfection, cells were stimulated with ET-1 together or not with macitentan. 24 h later, luciferase promoter activities were determined through the One-Glo luciferase assay system (Promega, #E6120) on the GloMax 96 Microplate Luminometer (Promega). The activity of β-galactosidase, determined by using the Chlorophenolred-β-D-galactopyranoside (CPRG) substrate (Roche, #10884308001), was employed to normalize the luciferase activity of the reporter constructs.

### Chromatin immunoprecipitation assay

Chromatin immunoprecipitation (ChIP) experiments were performed by using chromatin extracted from 5 × 10^6^ cells of PD HG-SOC or Kuramochi cells as previously reported [15] and by using anti-ZEB1 (2 µg, Santa Cruz Biotechnology, #sc-515797), anti-ZEB2 (2 μg, Santa Cruz Biotechnology, #sc-271984), anti-GSDME (5 µg, Abcam, #ab215191), anti-mouse IgG isotype control (2 μg, Thermo Fisher Scientific) or anti-rabbit IgG isotype control (5 μg, Thermo Fisher Scientific) Abs. The recruitments of GSDME and/or ZEB1 and ZEB2 proteins in the GSDME, E-cadherin, vimentin, and IL-6 promoter sequences were assessed through qPCR, by employing the AmpliTaq polimerase (Applied Biosystems, USA, #N8080156) and the GeneAmp PCR system 9700 (Applied Biosystems). The primers used are listed in Supplementary Information, Table S3.

### Proximity ligation assay

PD HG-SOC cells (4 × 10^4^) were seeded into a 24-well plate on glass coverslips. After 24 h of starvation, cells were stimulated or not for 12 h with ET-1 in the presence or not of macitentan. Proximity ligation assay (PLA) was carried out as previously described [15] and by using anti-ZEB1 (H-3, 1:10, Santa Cruz Biotechnology, #sc-515797) together with anti-GSDME (1:40, Abcam, Cambridge, UK, #ab215191) primary Abs. 4′,6′-diamidino-2-phenykindole (DAPI, BioRad, #1351303) was utilized to stain the nuclei and Vectashield medium (Vector Laboratories Ltd., Manchester, UK, #H1000) was employed to mount the coverslips. Images were taken with the DMIRE2 microscope (Leica, Wetzlar, Germany) at 63× objective. The number of dots per nuclei was quantified through the ImageJ software.

### Proteome profiler array

PD HG-SOC cells (1 × 10^6^) cells were seeded in 100 mm dish. After 24 h of siRNA transfection, cell medium was replaced with serum-free medium containing or not ET-1 alone or in combination with macitentan for 48 h. Conditioned media (CM) were then collected, centrifuged for 10 min at 1000 g and stored at -80°C. Proteome profiler assay was performed by using 50 μl of CM and the human cytokine array (R&D Systems, Minneapolis, MN, USA, #ARY005B), following the manufacturer’s instructions. Membranes were developed with a chemoluminescent reagent provided in the kit and the average signal (pixel density) of each spot was captured at equal exposure time with the ChemiDoc Image System.

### ELISA assay

To analyze the IL-6 release in CM from both PD HG-SOC and Kuramochi cells, we carried out Enzyme-Linked Immunosorbent (ELISA) assay by using the human Quantikine ELISA kit (R&D Systems, #D6050), as indicated by the manufacturer, and the Multiskan FC instrument (Thermo Fisher Scientific). IL-6 concentration (pg/ml) was determined in each sample by interpolating the absorbance values against the standard curve that was calculated by recombinant proteins at gradient dilution. Values were normalized to the number of cells at the end of the experiment and expressed as fold over control.

### Immunofluorescence analysis

PD HG-SOC cells were siRNA transfected for 24 h and then 3 × 10^4^ cells per condition were seeded upon round glass coverslips placed in 24-well plates and were stimulated with ET-1 in the presence or not of macitentan for 48 h. Immunofluorescence (IF) experiments were performed as previously reported [33]. Cells were incubated with anti-E-cadherin (1:100, GeneTex, Irvine, CA, USA, #GTX629691) or anti-vimentin (1:50, Cell Signaling Technology, #5741) Abs overnight at 4 °C. Next day, Alexa Fluor 488-labeled goat anti-mouse (1:250, Thermo Fisher Scientific, #A11001) or Alexa Fluor 594-labeled goat anti-rabbit (1:250, Thermo Fisher Scientific, #A11037) were added for 1 h for the detection of E-cadherin and vimentin, respectively. Nuclei were stained with DAPI and, after the coverslips’ mounting with the Vectashield medium, IF signals were captured through the DMIRE2 microscope and with an oil 63× objective.

### Cell migration assay

Cell migration assay was performed in a 24-well plate containing 8 μm size Thincert cell culture inserts (Greiner Bio-One, Frickenhausen, Germany, #662638). After 48 h of siRNA transfection, PD HG-SOC or Kuramochi cells (3 × 10^4^) were seeded in 200 μl of starved medium at the top of the chamber and ET-1 (the chemoattractant) in combination or not with macitentan was added to the bottom of the chamber in 700 μl of starved medium. After an overnight growth, the cells that remained in the upper part of the bucket were detached and cells migrated to the bottom part of the chamber were stained with the Three-Step stain set (Epredia, Breda, Netherlands, #3300), following the suggestions of the manufacturer. Several phase-contrast images were captured for each well by using the ZOE Fluorescent Cell Imaging System (BioRad) at 20× magnification.

### Three-dimensional HG-SOC spheroid sprouting assay

Cell sprouting of PD HG-SOC spheroids was analyzed by plating 24 h siRNA-transfected cells in 96-well ultra-low cluster U-shaped plate (Costar, Arlington, VA, USA, #7007; 100 cells/well). When spheroids are assembled, 50 μl/well of Cultrex reduced growth factor basement membrane extract (R&D Systems, #3433-005-01) was added and stimuli (ET-1 in the presence or not with macitentan) were applied for 48 h, as indicated. Spheroids were photographed in phase contrast before and after treatment by using the inverted ZEISS Axio Vert.A1 microscope at 10× magnification. ImageJ software was employed to quantify the length of the sprouts.

### Animal models and procedures

All animal care and experimental procedures were approved by IRCCS Regina Elena Cancer Institute Animal Care and Use Committee and the Italian Ministry of Health (D.lgs 26/2014, authorization number 1083/2020PR, issued 5 November 2020 by Ministero della Salute). Eight-week-old female nude-CD1 mice (Envigo Laboratories, Indianapolis, IN, USA) were housed under controlled temperature and humidity, following a 12 h light/dark cycle in pathogen-free conditions. Mice were intraperitoneally (i.p.) injected with 2 × 10^6^ viable PD HG-SOC cells or with 3 × 10^6^ viable of Kuramochi-Luc cells, previously infected with a luciferase-expressing lentiviral vector, to generate patient-derived xenografts (PDX) and Kuramochi xenografts, respectively. After 1 week of latency, mice were randomly divided into two groups (8 mice/group) and were treated through oral gavage with methocel (Ctr; vehicle; Sigma Aldrich) or macitentan (MAC; 30 mg/kg/daily). Mice that showed signs of distress were promptly euthanized according to the international animal welfare guidelines. Notably, macitentan treatment did not induce toxicity symptoms as body loss in both the experimental settings. In Kuramochi xenografts, tumor burden was measured through an in vivo imaging system (IVIS), once per week, as previously reported [33]. After five weeks, mice were euthanized and sacrificed by dislocation. Visible metastases were counted into the organs of peritoneal cavity. IB or qRT-PCR analyses were performed after the removing and processing of metastatic nodules. Values represent the mean ± SD of eight mice in each group for PDX and Kuramochi xenografts from two independent experiments.

### Bioinformatics analyses

Normalized gene expression of HG-SOC patients was taken by the Broad Institute The Cancer Genome Atlas (TCGA) Genome Data Analysis Center (2016): TCGA data from Broad GDAC Firehose 2016_01_28 run. Broad Institute of MIT and Harvard. Dataset. 10.7908/C11G0KM9. Differences of gene expression in the subgroups of patients from TCGA were analyzed. mRNA expression from Cancer Cell Line Encyclopedia (CCLE) was downloaded from cBioPortal [34] and selected for the following HG-SOC cell lines: CAOV3, COV318, COV362, FUOV1, HEYA8, JHOS2, JHOS4, Kuramochi, OAW28, OVCAR4, OVCAR8, OVSAHO, SNU119 and TYKNU. Association between genes was assessed by Pearson’s correlation. Basing on GSDME gene or ET_A_R/GSDME/ZEB1 gene signature, a risk score was evaluated for each HG-SOC patient from the TCGA or GSE9891 cohorts grouped in high/low risk basing on positive/negative risk scores, respectively. The risk score was calculated as: RS=Σ^*n*^_*i*=1_ βi_*_ *xi*, where *β* was the coefficient of the cox regression for the gene *i* and *x* is the gene expression. The model was applied for Kaplan-Meier (K-M) overall survival (OS) analyses. Gene set enrichment analysis (GSEA) was performed to explore signaling pathways associated with GSDME high-risk versus low-risk patients, using GSEA version 4.2 software from the Broad Institute, run in pre-ranked mode with hallmark gene sets. Pathways are sorted by False Discovery Rate and normalized enrichment score (NES).

### Statistical analysis

Except for the in vivo studies, each experiment was repeated in triplicate at least three times. Data points in the graphs represent the values of the means ± the standard deviation (SD) of three independent experiments. Statistical significance was defined by the GraphPad Prism 9 software through the two-tailed Student’s t-test, used for the comparison of two groups of independent samples, or through the one-way ANOVA test, used for multiple group comparison. Log-rank test was employed to assess the statistical significance of the distance between OS curves by using the MATLAB software. The sample size of mice chosen for each treatment group was calculated through the Student’s *t* test. *P* < 0.05 was considered the cut-off threshold of significance.

## Results

### ET-1/ET_A_R axis enhances the expression of GSDME in HG-SOC cells

**Fig. 1:**
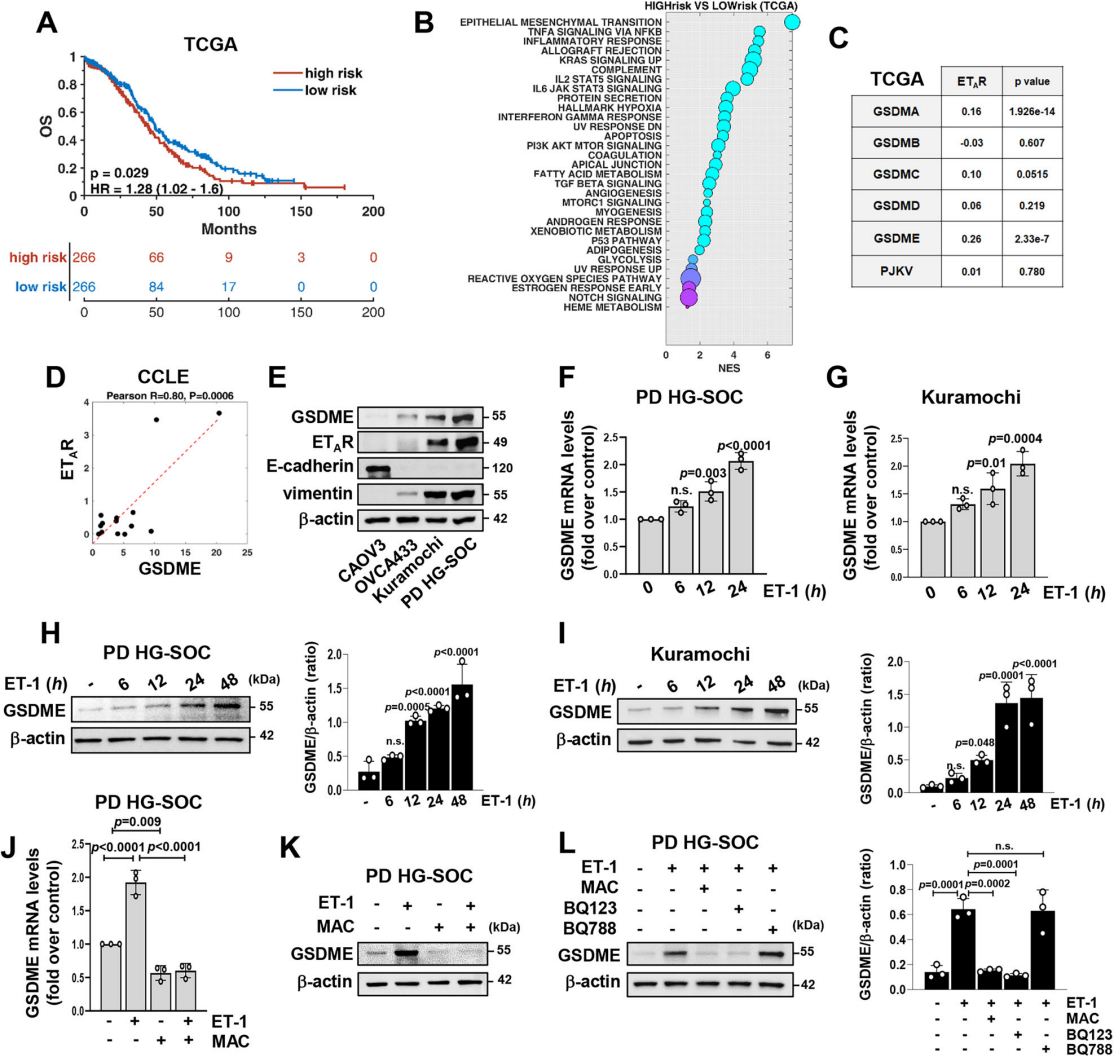
ET-1/ET_A_R axis promotes GSDME expression in HG-SOC cells. **A** Kaplan–Meier overall survival (OS) analysis of high-grade serous ovarian cancer (HG-SOC) patients from The Cancer Genome Atlas (TCGA) dataset showing high-risk score patients (*n* = 266) compared to low-risk score patients (*n* = 266) basing on GSDME gene expression. **B** Bubble plot depicting the gene set enrichment (GSEA) of hallmark pathways in the same high-risk group of HG-SOC patients analyzed in A. *X*-axis indicates the normalized enrichment score (NES), while circles’ dimension represents the percentage of genes included in the pathway. **C** Table showing Pearson’s correlation analysis between the mean gene expression of ET_A_R and each GSDM family member in HG-SOC patients from TCGA (*n* = 379). **D** Scatter plot for correlation estimation of GSDME and ET_A_R gene expression in HG-SOC cell lines from Cancer Cell Line Encyclopedia (CCLE) collection (*n* = 14). R indicates the Pearson correlation coefficient. **E** Immunoblotting (IB) analyses detecting GSDME, ET_A_R, E-cadherin and vimentin protein expression in whole cell extracts from patient-derived (PD) HG-SOC cells and HG-SOC cell lines (CAOV3, OVC433 and Kuramochi), as indicated. β-actin was employed as a loading control. **F,** **G** qRT-PCR analysis for GSDME gene expression in PD HG-SOC (**F**) and Kuramochi (**G**) cells stimulated with ET-1 (100 nM) for the indicated times. Values are the means ± SD normalized to cyclophilin-A and relative to the control (unstimulated cells), *n* = 3 independent experiments, n.s.= no significance. **H,** **I** IB analyses for the detection of GSDME protein expression in PD HG-SOC (**H**) and Kuramochi (**I**) cells stimulated with ET-1 for the indicated times. Right graphs show the densitometric quantification of GSDME/β-actin ratio. Values are the means ± SD, *n* = 3 independent experiments, n.s.= no significance. **J** qRT-PCR analysis assessing the mRNA expression of GSDME in PD HG-SOC cells stimulated for 24 h with ET-1 and/or with macitentan (MAC, 1 μM). Values are the means ± SD normalized to cyclophilin-A and relative to the control (unstimulated cells), *n* = 3 independent experiments. **K** GSDME protein expression evaluated through IB in total extracts of PD HG-SOC cells stimulated or not for 48 h with ET-1 and/or with macitentan. **L** IB analysis for GSDME expression in PD HG-SOC cells stimulated as in K, or with ET-1 in co-presence of the ET_A_R antagonist BQ123 (1 µM) or the ET_B_R antagonist BQ788 (1 µM). Right graph shows the densitometric quantification of GSDME/β-actin ratio. Values are the means ± SD, *n* = 3 independent experiments, n.s.= no significance.

Given that GSDME gene expression has been associated with EMT across distinct forms of cancer, including ovarian cancer [25], we evaluated the functional correlation of GSDME with the aggressive nature of HG-SOC. To this end, we firstly conducted Kaplan-Meyer analyses using HG-SOC patients from TCGA dataset [2]. We dichotomized patients into high- and low-risk groups, based on GSDME gene expression, ascertaining that high levels of this GSDM member were associated with a higher risk of poor OS (Fig. 1A; HR = 1.28 (1.02-1.6), *p* = 0.029). Notably, GSEA, performed on the high-risk score group of patients, identified EMT as the top hallmark pathway that positively associated with GSDME expression (Fig. 1B), strengthening the conceivable involvement of GSDME in the EMT process. To assess the specific relationship between each GSDM family member and EMT process in HG-SOC, we employed the EMTome source, a comprehensive EMT multiomics platform able to integrate data from EMT hallmark signature of Molecular Signatures database (MSigDB) with gene expression data from the TCGA cohort of HG-SOC [35, 36]. Our findings revealed a higher correlation of GSDME with the EMT compared to the other GSDMs (Supplementary Fig. S1A).

In search of regulatory factors impinging on GSDME expression in mesenchymal HG-SOC, we focused on ET-1/ET_A_R axis, given its critical involvement in EMT [10–15]. Correlation analyses in the TCGA cohort suggested GSDME as the GSDM family member most closely related to ET_A_R gene expression (Fig. 1C). Accordingly, we discovered a strong positive correlation of GSDME with ET_A_R gene expression in HG-SOC cell lines from CCLE (Fig. 1D) [37], prompting us to focus on this GSDM for subsequent experiments.

IB analyses in a panel of HG-SOC cell lines (CAOV3, OVCA433 and Kuramochi) and primary cultures of PD HG-SOC cells [30] confirmed that GSDME abundance is linked with mesenchymal traits of HG-SOC cells, similarly to ET_A_R expression (Fig. 1E). Remarkably, in cells stimulated with ET-1 at different time-points we observed a time-dependent increase of GSDME expression at both the mRNA (Fig. 1F, G) and protein (Fig. 1H, I) levels. Interestingly, ET-1-induced expression of GSDME was restrained by macitentan (Fig. 1J–L and Supplementary Fig. S1B, C), a non-selective ET_A_R/ET_B_R antagonist approved by regulatory agencies for the therapy of pulmonary arterial hypertension [10, 38–40]. Moreover, the use of specific inhibitors of ET_A_R (BQ123) or ET_B_R (BQ788), uncovered the peculiar involvement of ET_A_R in conveying ET-1-generated signals that promotes GSDME expression in HG-SOC (Fig. 1L). Thus, our results indicate that ET-1/ET_A_R axis is an important EMT cue able to induce GSDME expression in mesenchymal HG-SOC.

### ET-1 signaling induces GSDME transcription via ZEB1

**Fig. 2:**
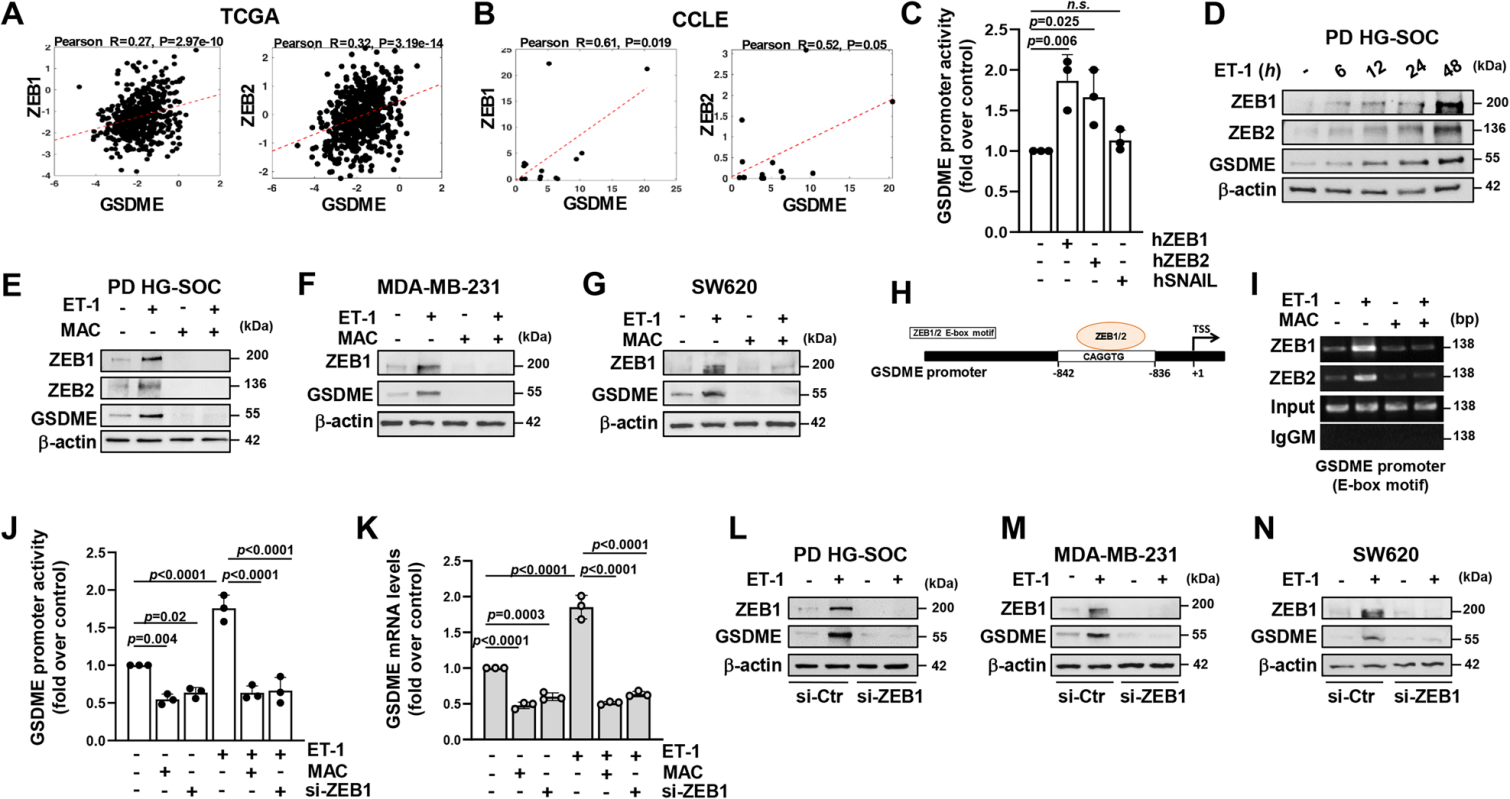
ET-1 signaling induces GSDME transcription via ZEB1. **A**, **B** Scatter plots showing the Pearson’s correlation between GSDME and ZEB1 or ZEB2 gene expression in HG-SOC patients (**A**) and HG-SOC cell lines (**B**) from the TCGA (*n* = 379) and CCLE (*n* = 14) databases, respectively. **C** Analysis of GSDME promoter luciferase activity performed in PD HG-SOC cells co-transfected for 24 h with a GSDME promoter reporter plasmid and with a control (Mock) plasmid or with human ZEB1 (hZEB1), ZEB2 (hZEB2), or SNAIL (hSNAIL) expression plasmids and stimulated or not with ET-1 for 24 h. Values are the means ± SD of data normalized to β-Gal and relative to control (unstimulated Mock cells), *n* = 3 independent experiments, n.s.= no significance. **D** IB analyses for ZEB1, ZEB2, and GSDME protein detection in PD HG-SOC cells stimulated with ET-1 for the indicated times. β-actin was employed as a loading control. **E**–**G** GSDME, ZEB1, or ZEB2 protein expression in PD HG-SOC cells (**E**), breast cancer MDA-MB-231 cells (**F**), and in SW620 colon cancer cells (**G**) stimulated for 48 h with ET-1 and/or with macitentan (MAC), assessed through IB. β-actin was employed as a loading control. **H** A schematic representation of the GSDME promoter sequence showing the distance of the E-box binding motif of ZEB1/2 to the transcription start site (TSS). **I** ZEB1/2 recruitment on their E-box binding motif of the GSDME promoter region in PD HG-SOC cells stimulated or not with ET-1 and/or with macitentan for 12 h, analyzed by chromatin immunoprecipitation (ChIP) assay followed by PCR. **J** Analysis of GSDME promoter reporter activity performed in PD HG-SOC cells co-transfected for 48 h with a GSDME promoter reporter plasmid and with a pool of siRNAs of control (si-Ctr) or with a pool of siRNAs specific for ZEB1 (si-ZEB1) and stimulated as in E for 24 h, as indicated. Values are the means ± SD of data normalized to β-Gal and relative to control (unstimulated si-Ctr-transfected cells), *n* = 3 independent experiments. **K** qRT-PCR analysis for GSDME gene expression in PD HG-SOC cells siRNA-transfected as indicated for 72 h and stimulated as in E for 24 h. Values are the means ± SD normalized to cyclophilin-A and relative to control (unstimulated si-Ctr-transfected cells), *n* = 3 independent experiments. **L–N** ZEB1 and GSDME protein expression assessed through IB in total extracts of siRNA-transfected PD HG-SOC (**L**), MDA-MB-231 (**M**), and SW620 (**N**) cells stimulated or not with ET-1 for 48 h. β-actin was employed as a loading control.

Because high levels of GSDME are associated with ZEB1 expression [25], we aimed to explore whether EMT-TFs could impact the regulation of GSDME expression. Pearson correlation analyses in TCGA cohort of HG-SOC patient and HG-SOC cell lines from CCLE database, identified a greater association between gene expression of GSDME and ZEB1 or ZEB2 compared to other core EMT-TFs, including SNAIL, SLUG and TWIST (Fig. 2A, B and Supplementary Fig. S2A, B). Consistently, luciferase experiments using a synthetic GSDME promoter, revealed a significant increase in the activity of this reporter in PD HG-SOC cells overexpressing ZEB1 or ZEB2, while a slight effect was observed in cells forced to express SNAIL (Fig. 2C and Supplementary Fig. S2C), implying an involvement of the ZEB family in the control of GSDME expression in HG-SOC cells. To explore the contribute of ET_A_R activation, we analyzed by IB the ability of ET-1 to induce ZEB1/2 and GSDME expression. ET-1 stimulation promoted a time-dependent increase in ZEB1 and ZEB2 protein expression with the same kinetic of GSDME (Fig. 2D and Supplementary Fig. S2D), which was hindered by the blockade of ET_A_R with macitentan (Fig. 2E and Supplementary Fig. S2E). Next, ChIP experiments in ET-1-stimulated PD HG-SOC cells unveiled the recruitment of ZEB1/2 on their canonical E-box binding domain (CAGGTG) in the GSDME promoter sequence that was inhibited by ET-1R blockade (Fig. 2H, I). The essential contribution of ZEB1 in the transcriptional regulation of GSDME driven by ET-1 signaling was proven by luciferase experiments with the GSDME promoter reporter in cells silenced or not for ZEB1 expression. In the absence of ZEB1, ET-1 stimulation lost its capacity to enhance the transcriptional activity of this promoter, likewise ET-1R inhibition by macitentan (Fig. 2J and Supplementary Fig. S2G). Accordingly, ET-1 signaling did not retain its ability to up-regulate either the mRNA and protein expression of GSDME in cells silenced for ZEB1 (Fig. 2K, L and Supplementary Fig. S2G–J).

Noteworthy, we corroborated the finding of GSDME fine-tuning by the ET_A_R/ZEB1 axis in breast (MDA-MB-231) and colon (SW620) cancer cell lines, both expressing ET_A_R (Supplementary Fig. S2F), underscoring the ability of ET-1 to upregulate GSDME in the absence of macitentan (Fig. 2F, G) and in a ZEB1-dependent manner (Fig. 2M, N). Overall, these findings point to the role of ET-1/ET_A_R axis in sustaining the expression of GSDME by inducing its transcription via ZEB1.

### ET-1 signaling regulates the assembly of a nuclear GSDME/ZEB1 transcriptional complex

**Fig. 3:**
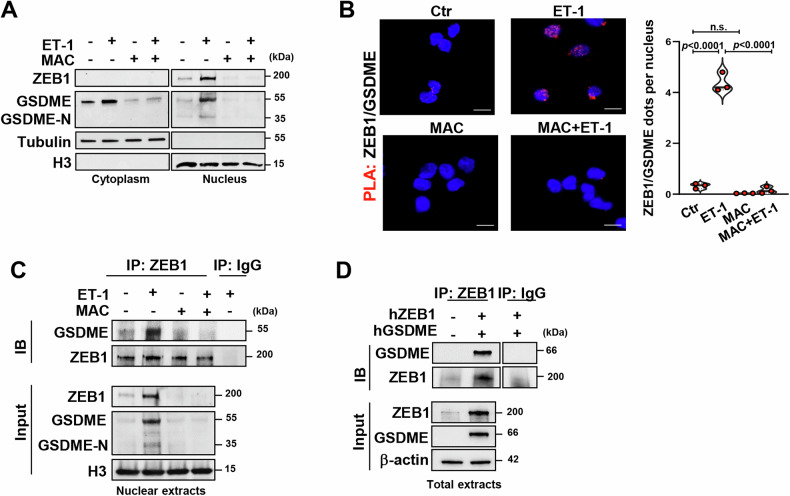
ET-1 signaling regulates the assembly of a nuclear GSDME/ZEB1 transcriptional complex. **A** IB analyses evaluating GSDME and ZEB1 protein expression in cytoplasmic and nuclear extracts of PD HG-SOC cells stimulated or not for 12 h with ET-1 in the presence or not of macitentan (MAC), as indicated. Tubulin and histone H3 (H3) were used as cytoplasmic and nuclear loading controls, respectively. **B** Proximity ligation assay (PLA) images revealing the ZEB1/GSDME protein interaction (red signals) in PD HG-SOC cells stimulated as in A. DAPI staining (blue signal) marks the nuclei (original magnification: 63x; scale bar: 10 μm). Right graph shows the quantification of the fluorescent red dots normalized to the number of nuclei. Values are the means ± SD, *n* = 3 independent experiments, n.s.= no significance. **C** Co-immunoprecipitation (co-IP) and IB analyses revealing the interaction between endogenous GSDME with ZEB1 proteins performed with nuclear lysates from PD HG-SOC cells stimulated as in A, IP with anti-ZEB1 or anti-immunoglobulin G (IgG) antibodies (Abs), and IB with anti-ZEB1 and anti-GSDME Abs. H3 was used as loading control. **D** co-IP and IB analyses detecting ZEB1 and GSDME interaction carried-out as in C by using total lysates of PD HG-SOC cells transfected for 24 h with a control plasmid or with hZEB1 and hGSDME. β-actin was employed as a loading control.

The full-length form of GSDME (GSDME) usually localizes in the cytoplasm where it is cleaved by caspase-3 in GSDME N-terminal fragment (GSDME-N) [17–21]. Considering the complexity of GSDM functions [17, 19, 41], we evaluated whether ET-1 signaling may affect GSDME subcellular localization in HG-SOC cells. In the nuclear compartments of ET-1-stimulated cells, we recognized, by employing an antibody able to detect both the full-length and the N-terminal forms of GSDME, the induction of both the GSDME and GSDME-N protein forms (Fig. 3A and Supplementary Fig. S3A). The specificity of IB signals was assessed in the nuclei of GSDME-silenced cells, where we observed a loss of the full-length and N-terminal forms of GSDME induced by ET-1 compared to control cells (Supplementary Fig. S3B, C), advising possible novel functions of GSDME driven by ET-1. Moreover, the treatment with ET-1R antagonist effectively constrained the nuclear content of both the full-length of GSDME and GSDME-N (Fig. 3A and Supplementary Fig. S3A).

Since a nuclear co-presence of GSDME and ZEB1 was ascertained following ET-1 stimulation (Fig. 3A and Supplementary Fig. S3A), we leveraged in situ PLA to evaluate whether these proteins may interact each other. We observed an enhanced number of fluorescent nuclear dots in cells stimulated with ET-1, while a weak signal was noted in the nuclear fraction of cells pre-treated with macitentan (Fig. 3B), implying the ET-1/ET_A_R axis role in guiding the nuclear GSDME/ZEB1 protein complex assembly. In line with these results, co-IP analyses with a ZEB1 antibody indicated the interaction of endogenous GSDME and ZEB1 proteins in the nuclear lysates from ET-1-stimulated cells that was restrained by ET-1R blockade with macitentan (Fig. 3C and Supplementary Fig. S3D). Accordingly, IP experiments in cells co-transfected with exogenous ZEB1 (hZEB1) and GSDME (hGSDME) expressing plasmids, confirmed the binding of GSDME and ZEB1 proteins (Fig. 3D). As such, our data point out that ET-1/ET_A_R axis regulates the concomitant nuclear localization of GSDME and ZEB1 favoring the GSDME/ZEB1 co-option into a nuclear complex.

### ET-1-induced nuclear GSDME/ZEB1 complex transcriptionally drives E-cadherin suppression

**Fig. 4:**
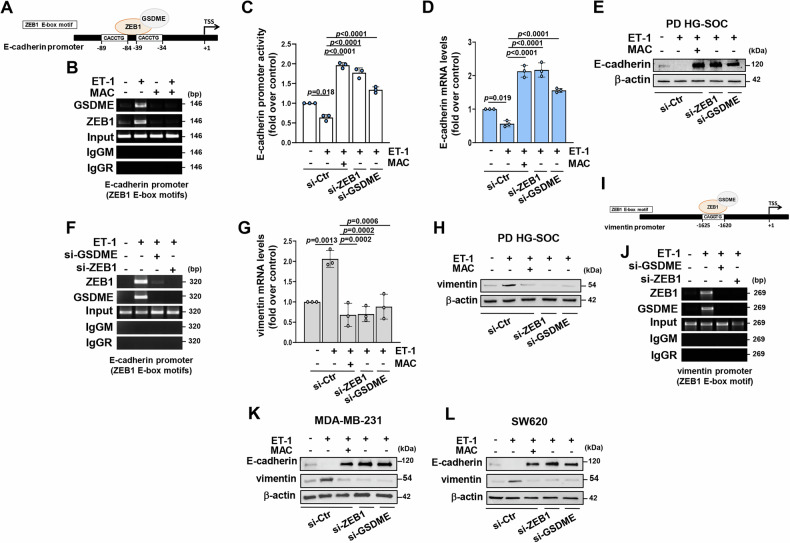
ET-1-induced nuclear GSDME/ZEB1 complex transcriptionally drives E-cadherin suppression. **A** Graphical representation of the proximal ZEB1 E-box binding motifs in the E-cadherin promoter sequence. **B** GSDME and ZEB1 recruitment on the E-cadherin promoter region containing binding sites for ZEB1 detected by ChIP assay followed by PCR in PD HG-SOC cells stimulated or not with ET-1 for 12 h in the presence or not of macitentan (MAC). **C** E-cadherin promoter luciferase activity measured in PD HG-SOC cells co-transfected with an E-cadherin promoter reporter plasmid and with si-Ctr, si-ZEB1 or with a specific pool of siRNAs for GSDME (si-GSDME) and stimulated as in B for 24 h, as indicated. Values are the means ± SD of data normalized to β-gal and relative to control (unstimulated si-Ctr-transfected cells), *n* = 3 independent experiments. **D** qRT-PCR for E-cadherin gene expression performed in siRNA-transfected PD HG-SOC cells stimulated as in C. Values are the means ± SD normalized to cyclophilin-A and relative to control (unstimulated si-Ctr-transfected cells), *n* = 3 independent experiments. **E** IB analysis assessing the E-cadherin protein expression in whole lysates from PD HG-SOC cells siRNA-transfected stimulated for 48 h as in C. β-actin was employed as a loading control. **F** ChIP assay for GSDME and ZEB1 recruitment on the E-cadherin promoter region in PD HG-SOC cells siRNA-transfected for 72 h as indicated and stimulated or not with ET-1 for 12 h. **G** Vimentin gene expression evaluated through qRT-PCR in PD HG-SOC cells transfected and stimulated as in D. Values are the means ± SD normalized to cyclophilin-A and relative to control (unstimulated si-Ctr-transfected cells), *n* = 3 independent experiments. **H** Vimentin protein expression assessed by IB analysis in whole lysates from PD HG-SOC cells transfected and stimulated as in E. β-actin was employed as a loading control. **I** Representation of ZEB1 E-box binding motif in the vimentin promoter sequence. **J** ChIP assay for GSDME and ZEB1 recruitment on the vimentin promoter region in PD HG-SOC cells transfected and stimulated as in (**F**). **K,** **L** IB analyses for E-cadherin and vimentin protein expression in whole lysates from MDA-MB-231 (**K**) or SW620 (**L**) cells transfected and stimulated as in E. β-actin was employed as a loading control.

In an attempt to elucidate the ET-1-driven functions of GSDME in HG-SOC, we examined whether this GSDM, as a nuclear interactor of ZEB1, may act as a regulator of EMT-related genes. Considering that the foremost function of ZEB1 is to suppress the transcription of E-cadherin for the advancement of the EMT program [8], we started from this gene to inquire into the possible transcriptional contribute of GSDME. We carried out ChIP experiments and analyzed the 5’ proximal promoter sequence of E-cadherin [42]. A recruitment of ZEB1 on its E-box binding motifs was detected upon ET-1 stimulation, along with the presence of GSDME in this specific E-cadherin promoter region, which was restrained by ET-1R blockade (Fig. 4A, B), suggesting a possible role of GSDME to act as a ZEB1 co-regulator in the repression of E-cadherin. To corroborate this assumption, HG-SOC cells were transfected with a reporter construct, enclosing the E-cadherin promoter region, and a reversion of ET-1-dependent repression of E-cadherin promoter activity was observed after GSDME silencing (Fig. 4C and Supplementary Fig. S4A), similarly ZEB1 depletion or ET_A_R inhibition (Fig. 4C and Supplementary Fig. S2G). Accordingly, GSDME exhaustion restrained the inhibitory effects of ET-1 signaling on E-cadherin mRNA (Fig. 4D) and protein (Fig. 4E and Supplementary Fig. S4A–C) expression, mimicking the effect of macitentan treatment or ZEB1 silencing (Fig. 4D, E and Supplementary Fig. S2G, H, S4C). Furthermore, ChIP assays in ET-1-stimulated and ZEB1-silenced or GSDME-depleted cells showed the complete abrogation of GSDME/ZEB1 recruitment to the previously analyzed E-cadherin promoter sequence (Fig. 4F and Supplementary Fig. S2G, H, S4A, B, D). Conversely, we observed a reduction in ET-1-induced vimentin expression at both the mRNA (Fig. 4G) and protein (Fig. 4H and Supplementary Fig. S2G, S4A) levels when HG-SOC cells were silenced for GSDME or ZEB1 or inhibited for ET_A_R. Furthermore, silencing of ZEB1 and GSDME in ChIP assay, revealed that even in the case of vimentin, these two transcriptional partners mutually control their recruitment to the promoter sequence containing an E-box binding site for ZEB1 induced by ET-1 (Fig. 4I, J). Similarly, the evidence of ZEB1/GSDME involvement in the regulation of EMT markers upon ET_A_R activation in breast and colon cancer (Fig. 4K, L and Supplementary Fig. S4E, F), suggested that this novel EMT-related nuclear function of GSDME may be common to various tumor histotypes, in addition to HG-SOC. Collectively, these data underscore a novel role for GSDME, which acts as an ET-1-induced co-regulator of ZEB1 to modulate target genes associated with the EMT program, as E-cadherin and vimentin.

### GSDME transcriptionally supports ET-1-induced inflammatory cytokine release

**Fig. 5:**
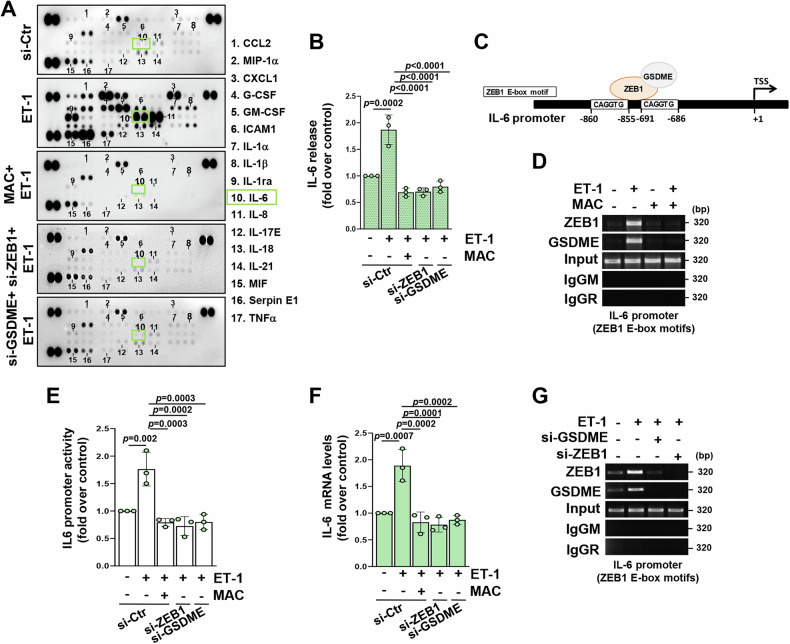
GSDME transcriptionally supports ET-1-induced inflammatory cytokine release. **A** Proteome profiling of cytokines detected in conditioned media (CM) from PD HG-SOC cells transfected for 72 h with specific siRNAs for ZEB1, GSDME or with control siRNAs and stimulated or not with ET-1 in the presence or not of macitentan (MAC) for 48 h. **B** ELISA assay measuring the IL-6 release in CM from PD HG-SOC cells transfected and stimulated as in A. Values are the means ± SD relative to control (unstimulated si-Ctr-transfected cells), *n* = 3 independent experiments. **C** Schematic representation of ZEB1 E-box binding motifs in the IL-6 promoter sequence. **D** Engagement of the ZEB1/GSDME protein complex on the IL-6 promoter region including ZEB1-binding motifs, examined by ChIP assay followed PCR in PD HG-SOC cells stimulated or not with ET-1 for 12 h and/or with macitentan. **E** Luciferase assay measuring the IL-6 promoter luciferase activity in PD HG-SOC cells co-transfected for 48 h with an E-cadherin promoter reporter plasmid and with indicated siRNAs and stimulated or not with ET-1 for 24 h in the presence or not of macitentan. Values are the means ± SD normalized to β-gal and relative to control (unstimulated si-Ctr transfected cells), *n* = 3 independent experiments. **F** IL-6 gene expression assessed by qRT-PCR in PD HG-SOC cells transfected as in A and stimulated as indicated for 24 h. Values are the means ± SD normalized to cyclophilin-A and relative to control (unstimulated si-Ctr transfected cells), *n* = 3 independent experiments. **G** ChIP assay assessing the GSDME/ZEB1 protein complex recruitment in the IL-6 promoter sequence in PD HG-SOC cells 72 h silenced or not for GSDME or ZEB1 expression and stimulated or not with ET-1 for 12 h.

In view of the role of ET-1/ET-1R axis in boosting inflammatory signals [10, 30, 40, 43], we performed a proteome profiling analysis on the secretome of HG-SOC cells, finding a plethora of cytokines upregulated upon ET-1 and restrained by the ET-1R blockade (Fig. 5A). In line with the emerging contribution of ZEB1 in inflammatory responses driving cancer progression [44, 45], we observed a reduction in the ET-1-induced inflammatory cytokine pattern in cells silenced for ZEB1 or GSDME expression (Fig. 5A), suggesting an overlapping influence of GSDME and ZEB1 on ET-1-driven cytokine release. Among the cytokines most highly regulated by the ET-1/ET_A_R-activated GSDME/ZEB1 signaling network, we noted interleukin (IL)-6, a gatekeeper of cancer cell/TME communication implicated in ovarian cancer growth, metastasis and immune evasion [46]. Blockade of the ET_A_R circuit, by macitentan treatment or silencing of ZEB1 or GSDME, specifically impaired the levels of IL-6 release in the CM from ET-1-stimulated cells (Fig. 5B and Supplementary Fig. S2G, H, S4A, B, S5). To assess whether ET-1-regulated IL-6 could be a transcriptional target of the GSDME/ZEB1 axis, we performed ChIP experiments, uncovering an engagement of both GSDME and ZEB1 proteins on the IL-6 promoter after the ET-1 stimuli that was hindered by the ET-1R blockade (Fig. 5C, D). In line with these findings, luciferase assays using a vector containing the IL-6 promoter sequence harboring the ZEB1 E-box motifs, unveiled an increased IL-6 transcriptional promoter activity in response to ET-1 that was constrained through the knockdown of GSDME or ZEB1, or by macitentan treatment (Fig. 5E and Supplementary Fig. S2G, S4A). Moreover, the depletion of ZEB1 and GSDME dampened the expression levels of IL-6 mRNA levels even under ET-1 stimulation (Fig. 5F and Supplementary Fig. S2G, S4A), similarly to ET-1R blockade. These results highlighted that the co-presence of GSDME/ZEB1 is an important requirement for the chromatin recruitment of this protein complex to the IL-6 promoter, driven by ET-1 (Fig. 5G and Supplementary Fig. S2G, S4A). Taken together, these results point to the GSDME/ZEB1 alliance as a key signaling hub for ET-1-activated transcriptional pathways associated with the release of inflammatory cytokines, above all IL-6, that is involved in the intricate interplay with the TME.

### GSDME is intertwined with the ET-1/ET_A_R axis to mediate HG-SOC aggressiveness

**Fig. 6:**
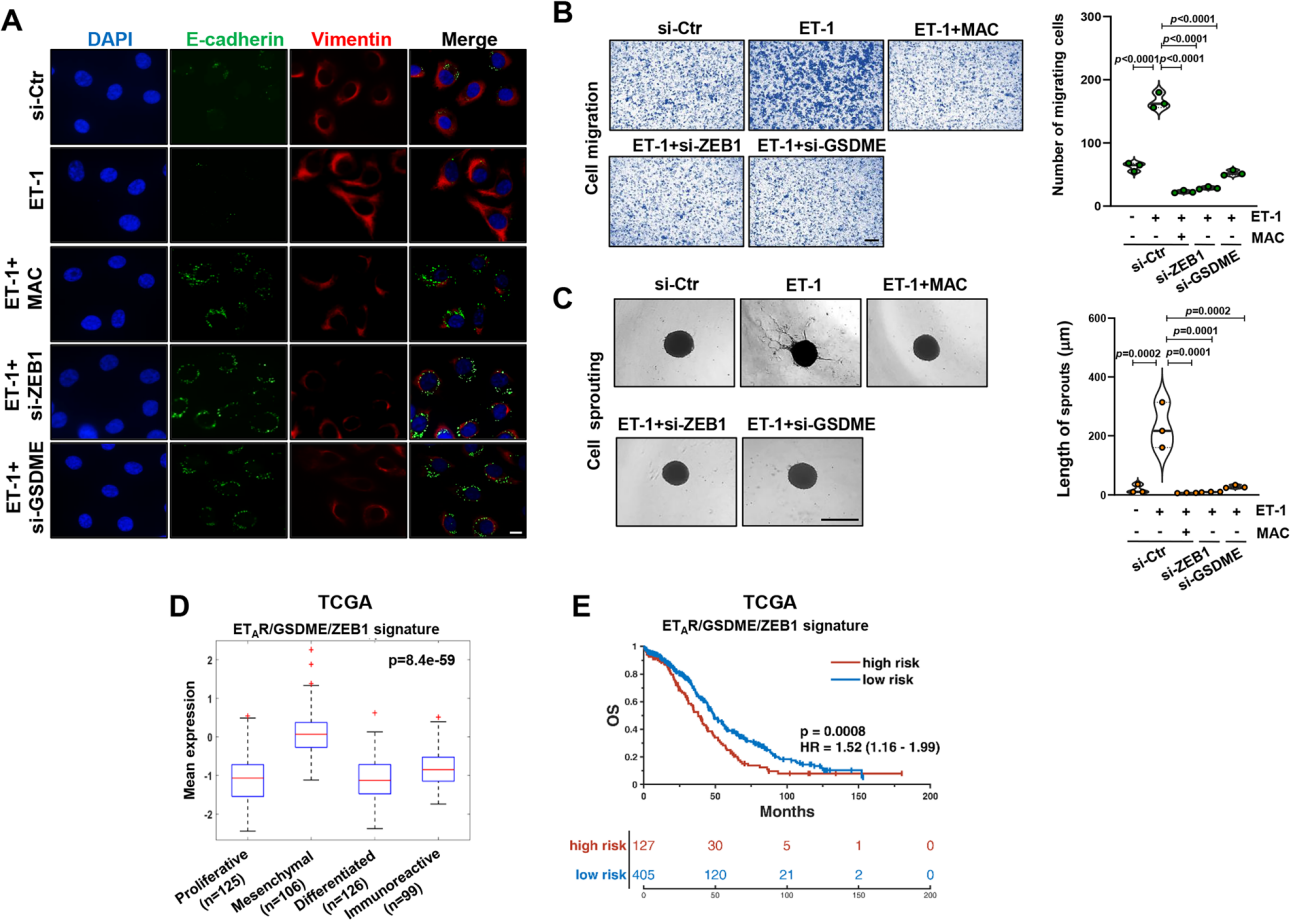
GSDME is intertwined with the ET-1/ET_A_R axis to mediate HG-SOC aggressiveness. **A** Representative images of immunofluorescence (IF) assay detecting E-cadherin (green signal) and vimentin (red signal) expression in PD HG-SOC cells transfected for 72 h with si-Ctr, si-ZEB1 or si-GSDME as indicated and stimulated or not with ET-1 for 48 h in the presence or not of macitentan (MAC). DAPI staining (blue signal) highlights the nuclei (magnification: 63x; scale bar: 10 μm). **B** Transwell migration assay of PD HG-SOC cells transfected as in A and overnight allowed to migrate in presence or not of ET-1 or macitentan as indicated. Images represent the crystal violet-stained migrated cells (magnification: 20x; scale bar: 100 μm). Right graph represents the number of migrating cells. Values are the means ± SD, *n* = 3 independent experiments. **C** Representative images of the sprouting of 3D spheroids of PD HG-SOC cells transfected as in A into the surrounding matrix upon stimulation for 48 h with ET-1 with or without macitentan (magnification 10x; scale bar: 100 µm). Right graph represents the quantification of cumulative sprout length (µm). Values are the means ± SD, *n* = 3 independent experiments. **D** Boxplot diagrams representing the expression of the ET_A_R/GSDME/ZEB1 molecular signature in HG-SOC patients from TCGA, classified in four transcriptional subtypes, as indicated. **E** Kaplan–Meier overall survival (OS) analysis of HG-SOC patients from TCGA dataset showing high-risk score patients (*n* = 127) compared to low-risk score patients (*n* = 405) based on the ET_A_R/GSDME/ZEB1 signature expression.

Having identified GSDME as an important factor for EMT-related gene transcription, we evaluate whether this GSDM may represent an advantage in ET-1-driven HG-SOC metastatic progression. Remarkably, IF analysis for E-cadherin and vimentin expression further supported that the presence of GSDME, similarly to ZEB1, is an important requisite for ET-1 to trigger a mesenchymal phenotype in PD HG-SOC (Fig. 6A and Supplementary Fig. S2G, S4A). In light of this evidence, we observed an enhanced migratory ability of cells after ET-1 stimulation that was curtailed by GSDME depletion, similarly to ZEB1 silencing and ET-1R inhibition (Fig. 6B and Supplementary Fig. S2G, H, S4A, B, S6A). In parallel, we performed 3D Matrigel invasion assay that showed the capacity of PD HG-SOC cells to invade across a surrounding matrix upon the chemotactic signal of ET-1 (Fig. 6C). Importantly, in cells depleted for GSDME expression a reduced invasion was remarked in response to ET-1, likewise to what observed in cells silenced for ZEB1 or treated with macitentan (Fig. 6C and Supplementary Fig. S2G, S4A), suggesting a coordinated activity of GSDME with the ET_A_R/ZEB1 axis in sustaining EMT plasticity, cell migration and invasion ruled by ET-1 signaling in HG-SOC cells.

We explored the predictive significance of ET_A_R/GSDME/ZEB1 integration, observing a marked expression of this molecular signature in the mesenchymal transcriptional cluster (Fig. 6D; *p* = 8.4e–59), a subgroup of patients exhibiting the worse clinical outcomes compared to the other clusters [2]. Importantly, in the cohort of TCGA patients, we ascertained an increase in the predictive power of survival risk by integrating GSDME with the ET_A_R/ZEB1 signature (Fig. 6E; HR = 1.52 (1.16-99), *p* = 0.0008) compared to GSDME alone (Fig. 1C). The prognostic relevance of ET_A_R/GSDME/ZEB1 signature was proved in another HG-SOC dataset, the GSE9891 cohort [1] (HR = 2.04 (1.38-3.01), *p* = 0.0001) (Supplementary Fig. S6B). Overall, these results demonstrate that GSDME integration with the ET_A_R/ZEB1 axis contributes to the acquisition of ET-1-induced aggressive traits in HG-SOC and suggest its potential for clinical predictions.

### ET-1R blockade impairs GSDME abundance and metastatic process in vivo

**Fig. 7:**
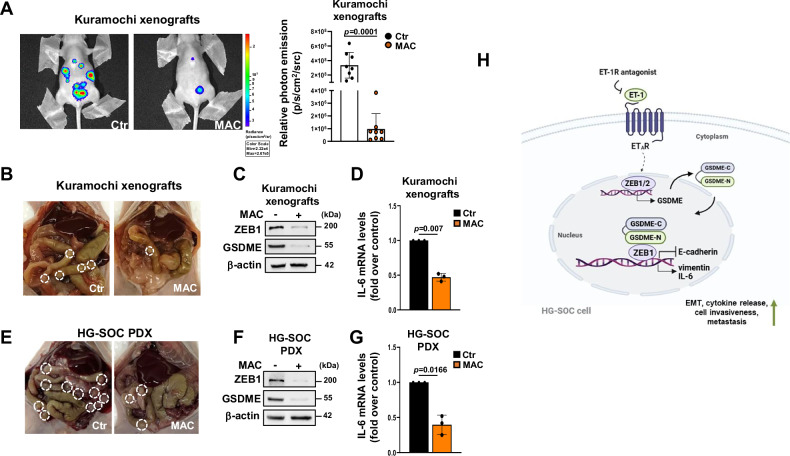
ET-1R blockade impairs GSDME abundance and metastatic process in vivo. **A** Representative bioluminescent images of i.p. Kuramochi-Luc-injected female mice (Kuramochi xenografts) treated with vehicle (Ctr) or macitentan (MAC, 30 mg/kg oral daily) for 5 weeks undergoing treatments (8 mice/treatment groups). Right graph represents the relative photon emission of the tumor burden at the end of the experiments. Values are the means ± SD for each treatment condition. **B**, **E** Representative images of intraperitoneal (i.p.) metastatic nodules (white circles) in the peritoneal cavity of Kuramochi xenografts (**B**) or HG-SOC PDX (**E**) treated as in A. **C**, **F** IB analyses assessing the expression of ZEB1 and GSDME proteins in total extracts from i.p. nodules of Kuramochi xenografts (**C**) or HG-SOC PDX (**F**) treated as in A. β-actin was employed as a loading control. **D**, **G** IL-6 gene expression funded out by qRT-PCR analyses in the same i.p. nodules of C and F from Kuramochi xenografts (**D**) or HG-SOC PDX (**G**). Values are the means ± SD normalized to cyclophilin-A and relative to control (Ctr mice), *n* = 3 independent experiments. **H** A schematic diagram illustrating the potential mechanism by which ET_A_R drives GSDME signaling to promote metastatic traits in HG-SOC cells. ET_A_R activation by ET-1 increases ZEB1 expression which, in turn, promotes the transcription of GSDME. Then, GSDME translocates into the nucleus and engages ZEB1 in an active transcriptional complex that suppresses E-cadherin and induces vimentin and IL-6 transcription. GSDME, downstream to the ET-1/ET_A_R axis, promotes EMT, cell invasion, inflammatory cytokine release and metastatic dissemination. Macitentan, an ET-1R antagonist, prevents HG-SOC metastatic progression curbing the GSDME/ZEB1 transcriptional activity (drown with the help of BioRender.com).

To assess how ET-1/ZEB1 axis-induced GSDME affects the metastatic process in vivo, we generated bioluminescent HG-SOC xenografts and PDX by i.p. injecting in nude mice D-Luciferin-transfected Kuramochi cells (Kuramochi-luc) or PD HG-SOC, respectively. Following a latency time, mice received the oral treatment with vehicle or macitentan for 5 weeks. In keeping with the important contribution of ET-1 signaling to tumor growth and metastasis [30], we found a reduction of the tumor bioluminescent intensity in Kuramochi xenografts in which ET_A_R was blocked by macitentan (Fig. 7A). In parallel, a decreased number of metastatic nodules was noticed in the peritoneal cavity after the treatment with macitentan in both Kuramochi xenografts (Fig. 7B; Ctr mice mean value = 7.87 vs macitentan-treated mice mean value = 3.37; *p* = 3.39088e-06) and HG-SOC PDX (Fig. 7E; Ctr mice mean value = 15.75 vs macitentan-treated mice mean value = 5.5; *p* = 1.13e-09) models. Notably, in metastatic nodules from Kuramochi and HG-SOC PDX mouse models we uncovered the downregulation of both GSDME and ZEB1 proteins in macitentan-treated subgroups (Fig. 7C, F). In these metastatic nodules we also observed a reduction in IL-6 gene expression under macitentan treatment (Fig. 7D, G), validating in vivo that inhibiting ET-1R may hinder the activity of the integrated GSDME/ZEB1 transcriptional complex, thereby reducing the metastatic progression of HG-SOC.

Altogether, these findings strength the importance of the use of ET-1R antagonist as a promising therapeutic approach to hamper the GSDME signaling in metastatic HG-SOC, indicating a strong rationale for exploiting ET-1 therapeutics to improve clinical benefits of HG-SOC patients.

## Discussion

Deciphering the complexity of EMT and metastatic programs could be helpful for developing personalized treatment for HG-SOC patients. This study provides insights into the regulatory circuitries underlying the metastatic process in HG-SOC, by uncovering the role of the ET-1/ET_A_R axis in regulating GSDME expression and its novel pyroptosis-independent nuclear function that underpins the acquisition of aggressive traits.

The upstream regulators of GSDMs expression in cancer have not yet been fully defined. Our results reveal that, in this tumor model, GSDME expression significantly associates with EMT. Similar to a reported evidence in non-small cell lung cancer and pancreatic cancer cells [24, 25], we found that ZEB family members, key TFs regulating EMT [8, 47], are strong inducers of GSDMs. Nevertheless, our study, by placing the ZEB1/2-dependent control of GSDME abundance downstream of ET-1 signaling, which is aberrantly expressed in HG-SOC [14, 30], provides important translational hints, identifying the ET-1/ET_A_R axis as a novel vulnerability that could be blocked to balance the levels of GSDME.

Previous studies in HG-SOC have mainly focused on the role of GSDME in pyroptosis, triggered by various stimuli, as treatment with PARP inhibitors, which lead to caspase-mediated cleavage of GSDME [26–28]. Our findings lead to a paradigm shift of GSDME from a key effector of the pyroptotic cell death program to a functional executor of EMT under the control of ET-1 system. In HG-SOC cells, we recognized an unconventional nuclear localization of both the full length and N-terminal forms of GSDME upon ET-1 stimulation. Importantly, we underscored the role of GSDME in modulating the transcription of important genes associated with EMT. The presence of GSDME is a critical determinant for suppression of E-cadherin and induction of vimentin expression, in an alliance with ZEB1 instructed by ET-1. These discoveries expand previous observations describing pyroptosis-independent effects of GSDM family members [22, 23, 48, 49], as the role of GSDMC in inducing genes related to stemness and immune evasion in pancreatic cancer [24].

Our mechanistic investigations reveal the functional significance of GSDME in ET-1-driving inflammatory responses in HG-SOC. We demonstrated that GSDME contributes in modulating the release of a broad range of ET-1-induced inflammatory cytokines. In particular, we identified IL-6 as a transcriptional target of the GSDME/ZEB1 complex downstream of the ET-1/ET_A_R axis. Given the role of this cytokine in HG-SOC [50–52], our findings imply a potential involvement of this transcriptional circuit in directing the crosstalk between cancer cells and components within the TME, as well as the role of its targeting in interrupting this cell-cell communication. Therefore, in our tumor model GSDME becomes a pivotal factor that increases cytokine abundance, supporting ZEB1-induced transcriptional control underlying the functional tumor/stroma interaction orchestrated by ET-1 for HG-SOC metastasis development.

Hence, we hypothesize that, following ET-1 stimulation, GSDME participates in a self-reinforcing circuit in which ZEB1 promotes GSDME expression to amplify EMT and the advancement of metastatic process by exploiting the transcriptional partnership with nuclear GSDME. We propose the use of macitentan as a promising therapeutic strategy able to target the ET_A_R-dependent transcriptional activity of GSDME, impairing the metastatic traits in cancer cells and the release of inflammatory cytokines, especially IL-6, one of the critical mediators of cross-compartment interaction. Of note macitentan, a dual ET_A_R/ET_B_R antagonist, may also obstacle the tumor progression targeting cells within the TME expressing both ET_A_R and ET_B_R, including stromal and immune cells [10, 40], which makes it an alternative treatment against metastatic dissemination in HG-SOC. Furthermore, our findings, revealing the ability of the ET_A_R/ZEB1 axis to control EMT-associated GSDME functions in breast and colon cancer cells, suggest exploring ET_A_R blockade to counteract nuclear transcriptional activities and metastatic progression in other tumor histotypes overexpressing ET_A_R.

Analyses on public cohorts of HG-SOC patients confirmed the clinical relevance of our results. We have demonstrated that the ET_A_R/GSDME/ZEB1 molecular signature represents a risk factor for poor overall survival, suggesting that ET-1-driven GSDME engagement in this transcriptional circuit contributes to the poor outcome of those patients who possess limited therapeutic benefit.

In conclusion, our results argue how GSDME becomes an integral part of the ET-1/ET_A_R molecular network that controls ZEB1-dependent transcriptional outputs underlying the metastatic process and associates with a poor clinical outcome of HG-SOC patients. Moreover, we suggest the leverage of the dual ET-1R antagonist macitentan to hinder the downstream GSDME-related transcriptional machinery activated by the ET-1/ET_A_R axis. At the mechanistic level, ET-1/ET_A_R signaling promotes the translocation of GSDME into the nucleus to regulate genes associated with EMT and inflammation (Fig. 7H). Thus, GSDME emerges as a critical regulator of ZEB1-mediated HG-SOC aggressiveness and suggest ET-1R targeting as an innovative approach to restrain the nuclear functions of GSDME and relieve the clinical outcomes of metastatic HG-SOC patients.

## Supplementary information

Supplementary Information

Original western blots

## Data Availability

The datasets generated and/or analyzed during the current study are available from the corresponding author on reasonable request.
