# Supplementary material for: Nuclear gasdermin E drives endothelin-1-induced metastatic progression independently of the pyroptosis
Source: Cell Death Dis. 2026 Jan 16;17(1):45. doi: 10.1038/s41419-025-08202-x (PMC12811335; doi:10.1038/s41419-025-08202-x)
Supplement: Supplementary file 1 — Supplementary Information [file 41419_2025_8202_MOESM1_ESM.pdf]

Supplementary Information

Nuclear gasdermin E drives endothelin-1-induced metastatic progression independently of the pyroptosis

Celia Roman, *et al.*

Table of contents

Supplementary Figure S1: 2

Supplementary Figure S2: 3

Supplementary Figure S3 5

Supplementary Figure S4: 6

Supplementary Figure S5: 7

Supplementary Figure S6: 8

Supplementary Table S1: 9

Supplementary Table S2: 9

Supplementary Table S3: 10

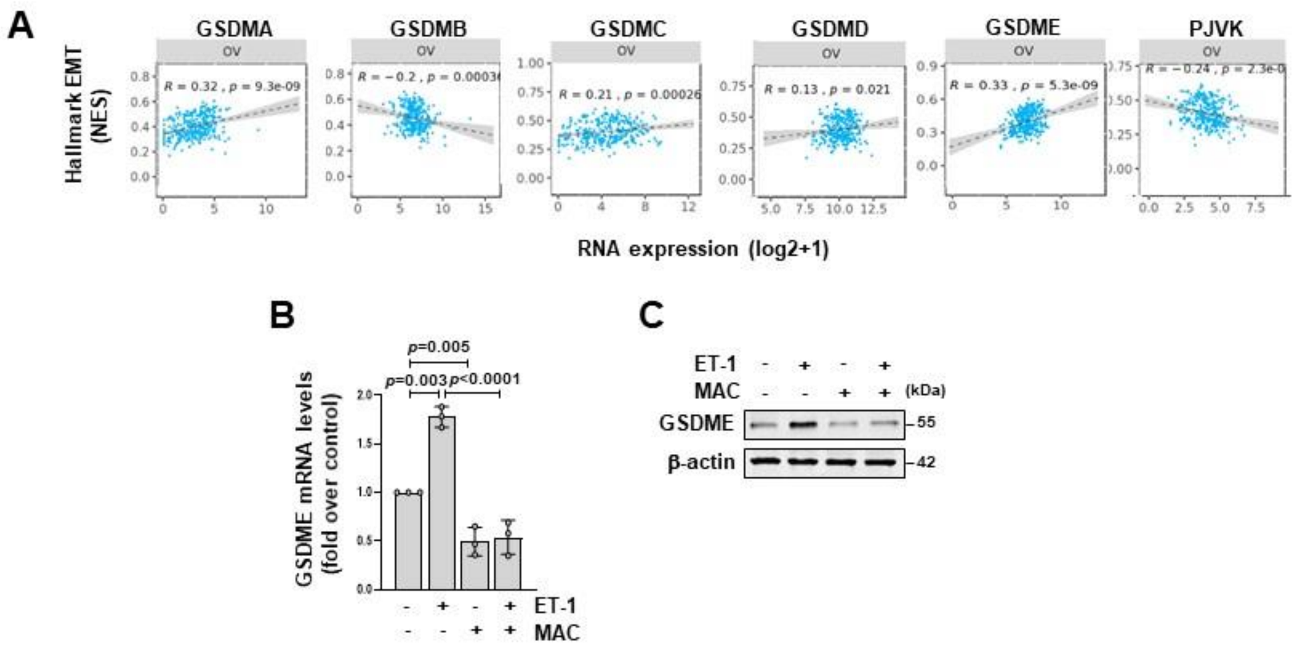

Supplementary Figure S1. GSDME expression associates with EMT and is induced by ET-1/ET$_A$R axis in HG-SOC. A Scatter plots for correlation estimation of gene expression of gasdermin (GSDM) members (GSDMA-E and Pejvakin) and hallmark EMT ssGSEA scores in the Cancer Genome Atlas (TCGA) dataset of high-grade serous ovarian cancer (HG-SOC) patients (*n*=602), generated through the EMTome source (emtome.org). R indicates Pearson correlation coefficient. **B** qRT-PCR in PD HG-SOC cells stimulated for 24 h with ET-1 (100 nM) and/or with macitentan (MAC, 1 μM) assessing the expression of GSDME at mRNA levels. Gene expression values are the means ± SD relative to control (unstimulated cells), *n*=3 independent experiments. **C** Immunoblot (IB) analysis detecting the protein expression of GSDME in PD HG-SOC cells stimulated as in B for 48 h. β-actin was used as loading control.

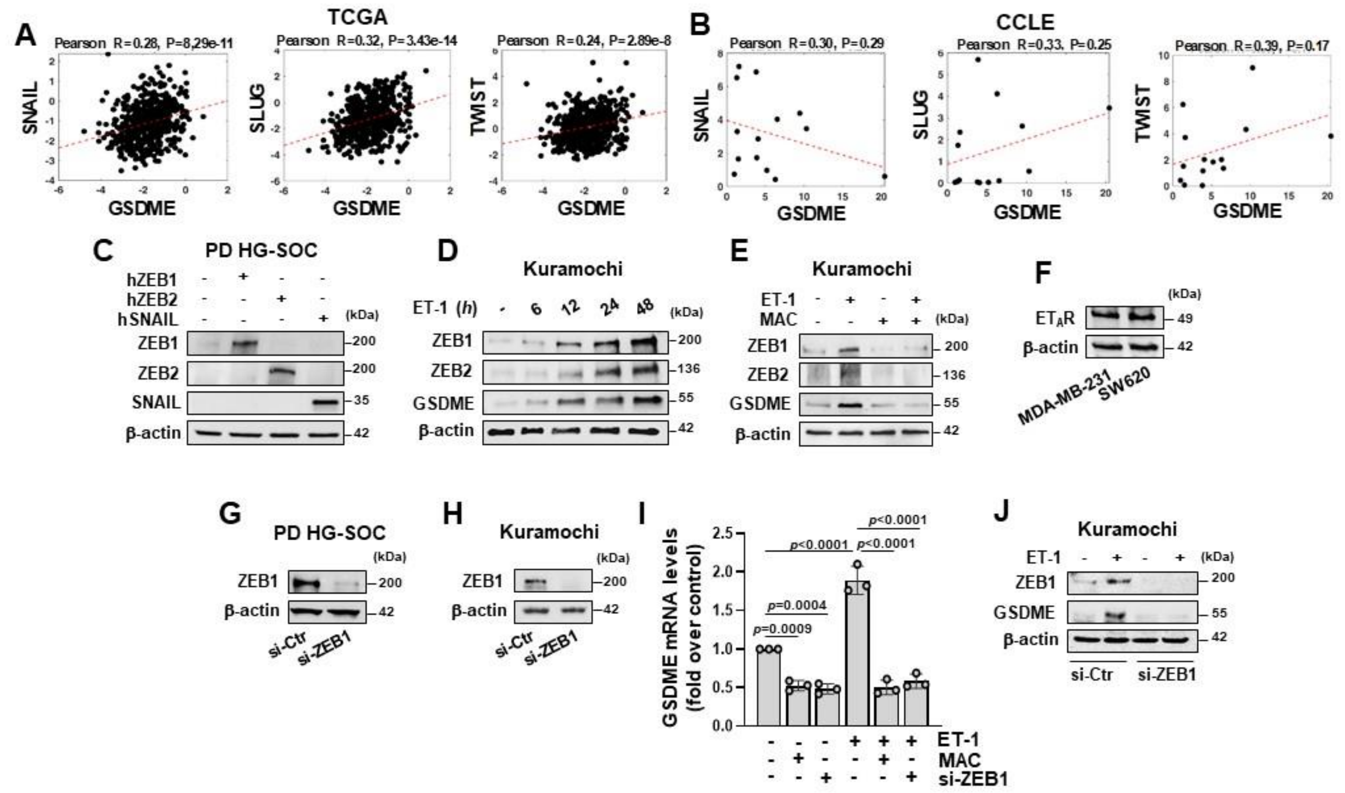

Supplementary Figure S2. ET-1/ET$_A$R axis induces the transcription of GSDME in a ZEB1-dependent manner. A, **B** Table showing Pearson's correlation analysis between the mean gene expression of GSDME with SNAIL, SLUG, or TWIST in HG-SOC patients from The Cancer Genome ATLAS (TCGA) dataset (n=379; **A**) or in HG-SOC cell lines from Cancer Cell Line Encyclopedia (CCLE) collection (n=14; **B**). R indicates Pearson correlation coefficient. **C** IB analysis in whole lysates from PD HG-SOC assessing the ectopic expression human flag-tagged ZEB1 (hZEB1), myc-tagged ZEB2 (hZEB2), or flag-tagged SNAIL (hSNAIL) after 24 h of transfection. β-actin was used as loading control. **D** ZEB1, ZEB2, and GSDME protein detection by IB in Kuramochi cells stimulated with ET-1 for the indicated times. β-actin was employed as a loading control. **E** IB analysis evaluating the expression of ZEB1, ZEB2, and GSDME proteins in Kuramochi cells stimulated with ET-1 and/or macitentan (MAC) for 48 h, as indicated. β-actin was used as loading control. **F** ET$_A$R protein expression analyzed through IB in breast cancer (MDA-MB-231) and in colon cancer (SW620) cells. β-actin was employed as a loading control. **G**, **H** ZEB1 silencing efficiency evaluated by IB in PD HG-SOC (**G**) and Kuramochi (**H**) cells transfected for 72 h with a

pool of siRNAs specific for ZEB1 (si-ZEB1) compared to cells transfected with a pool of non-targeting siRNAs (si-Ctr). **I** q-RT-PCR analysis for GSDME gene expression in Kuramochi cells siRNA-transfected as indicated for 72 h and stimulated as in E for 24 h. Values are the means ± SD normalized to cyclophilin-A and relative to control (unstimulated si-Ctr-transfected cells), n=3 independent experiments. **J** ZEB1 and GSDME protein expression assessed through IB in total extracts of siRNA-transfected Kuramochi cells stimulated or not with ET-1 for 48 h. β-actin was employed as a loading control.

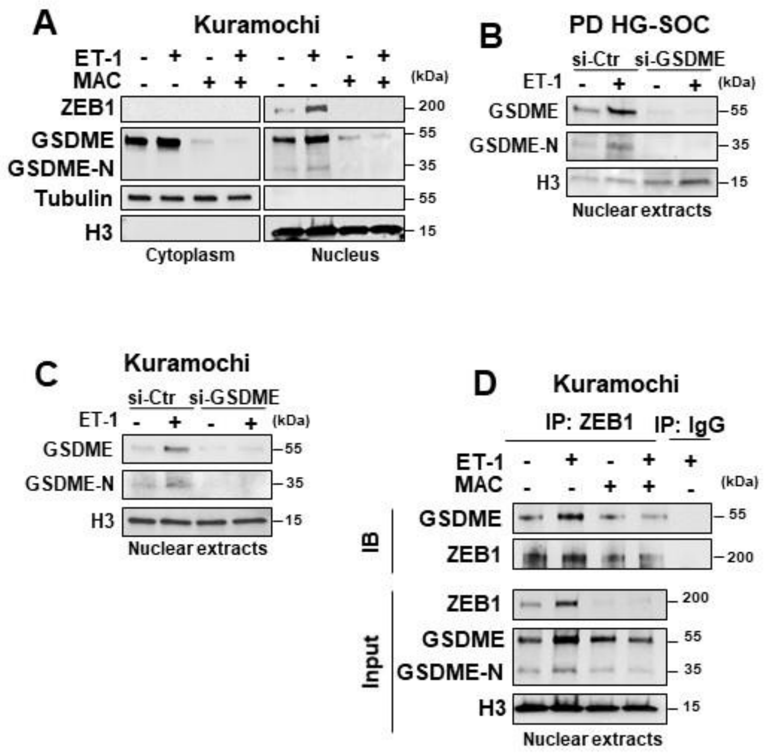

Supplementary Figure S3. ET-1/ET$_A$R axis promotes the engagement of a nuclear GSDME/ZEB1 complex. A IB analyses for ZEB1 and GSDME expression in the cytoplasmic and nuclear extracts of Kuramochi cells stimulated or not with ET-1 and/or with macitentan (MAC) for 12 h. Tubulin and histone H3 (H3) were used as cytoplasmic and nuclear loading controls, respectively. **B**, **C** IB analyses evaluating the efficiency of GSDME and GSDME-N silencing in the nuclear lysates from PD HG-SOC (**B**) and Kuramochi (**C**) cells transfected with a si-Ctr or with a pool of siRNAs specific for GSDME (si-GSDME) for 72 h and stimulated or not with ET-1 for 12 h. H3 was used nuclear loading control. **D** Nuclear extracts of Kuramochi cells stimulated as in A, immunoprecipitated (IP) for endogenous ZEB1, using anti-ZEB1 antibody (Ab) or anti-immunoglobulin G (IgG) Ab as control, and IB, using anti-ZEB1 and anti-GSDME Abs. H3 was used as loading control.

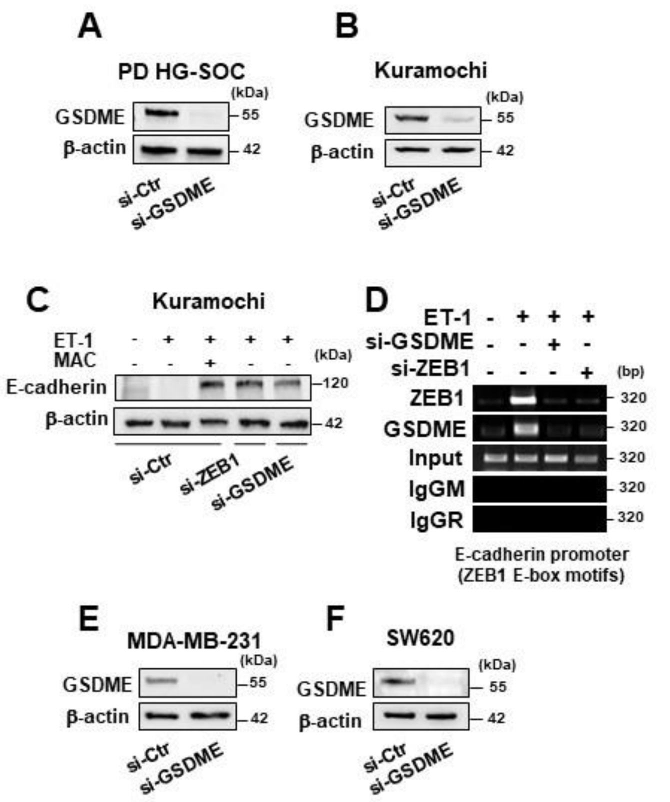

Supplementary Figure S4. GSDME is intertwined with the ET-1-ativated ET$_A$R/ZEB1 axis to reduce E-cadherin expression. A, **B** The efficiency of GSDME siRNAs in total extracts from PD-HG-SOC (**A**) or Kuramochi (**B**) cells after 72 h of transfection, evaluated through IB. β-actin was used as loading control. **C** E-cadherin protein expression in total extracts of Kuramochi cells siRNA-transfected for 72 h as indicated and stimulated or not with ET-1 and/or macitentan for 48 h analyzed by IB. β-actin was used as loading control. **D** GSDME and ZEB1 recruitment on the E-cadherin promoter region containing binding sites for ZEB1 detected by chromatin immunoprecipitation (ChIP) assay followed by PCR in Kuramochi cells transfected as indicated for 72 h and stimulated or not with ET-1 for 12 h. **E**, **F** IB analyses for GSDME expression in MDA-MB-231 (**E**) and SW620 (**F**) transfected for 72 h with control or GSDME siRNAs. β-actin was used as loading control.

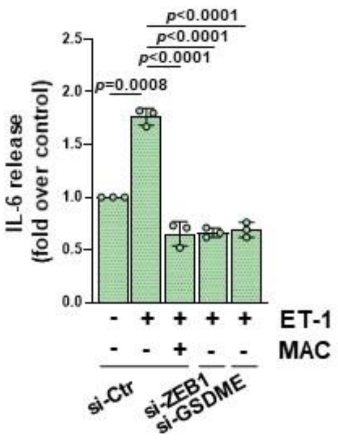

Supplementary Figure S5. GSDME depletion curbs the ET-1-triggered release of IL-6 similarly to ZEB1 inhibition and ET_AR blockade. ELISA assay detecting IL-6 release in conditioned media from Kuramochi cells transiently transfected for 72 h and stimulated for 48 h, as indicated. Values are the means ± SD normalized to the number of cells and relative to control (unstimulated si-Ctr-transfected cells), *n*=3 independent experiments.

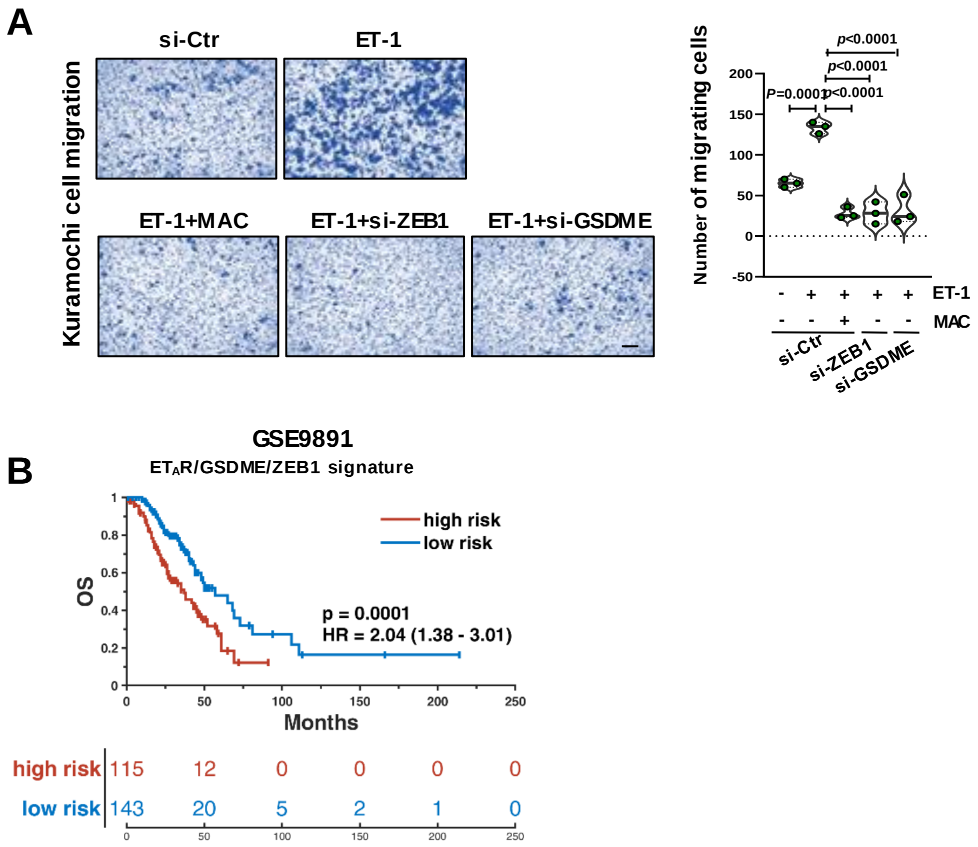

Supplementary Figure S6. The integrated ET$_A$R/ZEB1/GSDME signaling network mediates ET-1-driven HG-SOC cell migration and associates with a risk of poor survival in HG-SOC patients. A Transwell migration assay performed with Kuramochi cells siRNA-transfected as indicated for 72 h and overnight allowed to migrate in presence or not of ET-1 together or not with macitentan (MAC). Images represent the crystal violet-stained migrated cells (magnification: 20x; scale bar: 100 µm). Right graph represents the number of migrating cells. Values are the means ± SD, *n*=3 independent experiments. **B** Kaplan-Meier overall survival (OS) analysis of HG-SOC patients from GSE9891 dataset showing high-risk score patients (n=115) compared to low-risk score patients (n=143) based on the ET$_A$R/GSDME/ZEB1 signature expression.

Supplementary Table S1. Antibodies used in this study

Antigen	Dilution	Manufacturer
GSDME cat. #ab215191	1:1000	Abcam
ZEB1 (H-3) cat. #sc-515797	1:1000	Santa Cruz Biotechnology
ZEB2 (E6U7Z) cat. #97885	1:1000	Cell Signaling Technology
ET_AR cat. #PA3-065	1:3000	Thermo Fisher Scientific
E-cadherin (36) cat. #610181	1:500	BD Biosciences
Vimentin (D21H3) cat. #5741	1:1000	Cell Signaling Technology
SNAIL (L70G2) cat. #3895	1:1000	Cell Signaling Technology
β-actin (AC-15) cat. #1978	1:5000	Sigma Aldrich
Histone H3 (D1H2) cat. #4499	1:1000	Cell Signaling Technology
α-tubulin (DM1A) cat. #sc-32293	1:500	Santa Cruz Biotechnology

Supplementary Table S2. Primer sequences used for qRT-PCR experiments

Gene	Forward primer (5' to 3')	Reverse primer (5' to 3')
GSDME	CAGCCTACTTCTTGGTCAGTGC	TCTGTATCTTTCAGGGGAGTCA
E-cadherin	ACACCATCCTCAGCCAAGATCC	GTGGTGGGATTGAAGATCGGAG
Vimentin	TTTGAAGAAACTCCACGAAGAGGA	CCACATCGATTTGGACATGCT
IL-6	GGCACTGGCAGAAAACAACC	CACCAGGCAAGTCTCCTCAT
Cyclophilin-A	TTCATCTGCACTGCCAAGAC	TCGAGTTGTCCACAGTCAGC

Supplementary Table S3. Primer sequences used for ChIP experiments

Region	Forward primer (5' to 3')	Reverse primer (5' to 3')	Tm (°C)
GSDME promoter	GAGGCAAAGCTTAAATCTGTCTT	CAAGAGGGAAAGCTTTCTAATTG	58
E-cadherin promoter	ATGACGCGTGGCCGGCAGGTGAAC	ATGCTCGAGCGGGCTGGAGTCTGA	62
Vimentin promoter	ACACATTGAACGCTGTATATGTG	TTGCTTGAGATCAGGAGTTTGA	54
IL-6 promoter	AACCAAGTGGGCTTCAGTAATTT	CATCCTCAAATTTCGTGCAGTTA	58
