# Supplementary material for: Nuclear gasdermin E drives endothelin-1-induced metastatic progression independently of the pyroptosis
Source: Cell Death Dis. 2026 Jan 16;17(1):45. doi: 10.1038/s41419-025-08202-x (PMC12811335; doi:10.1038/s41419-025-08202-x)

Figure 1

Figure 1E

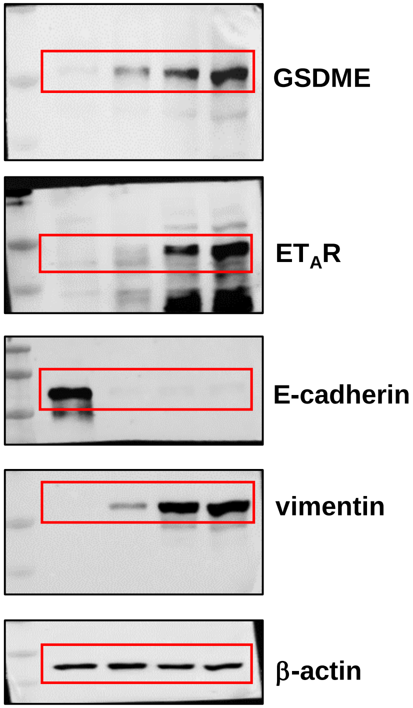

Figure 1H

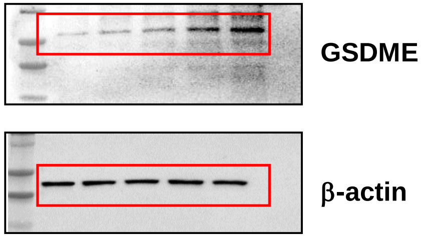

Figure 1I

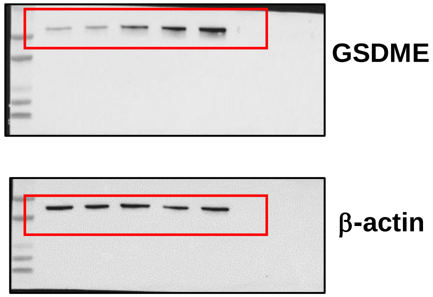

Figure 1K

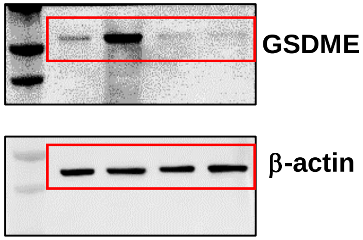

Figure 1L

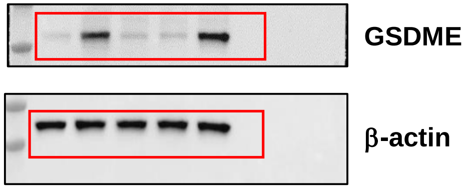

Figure 2

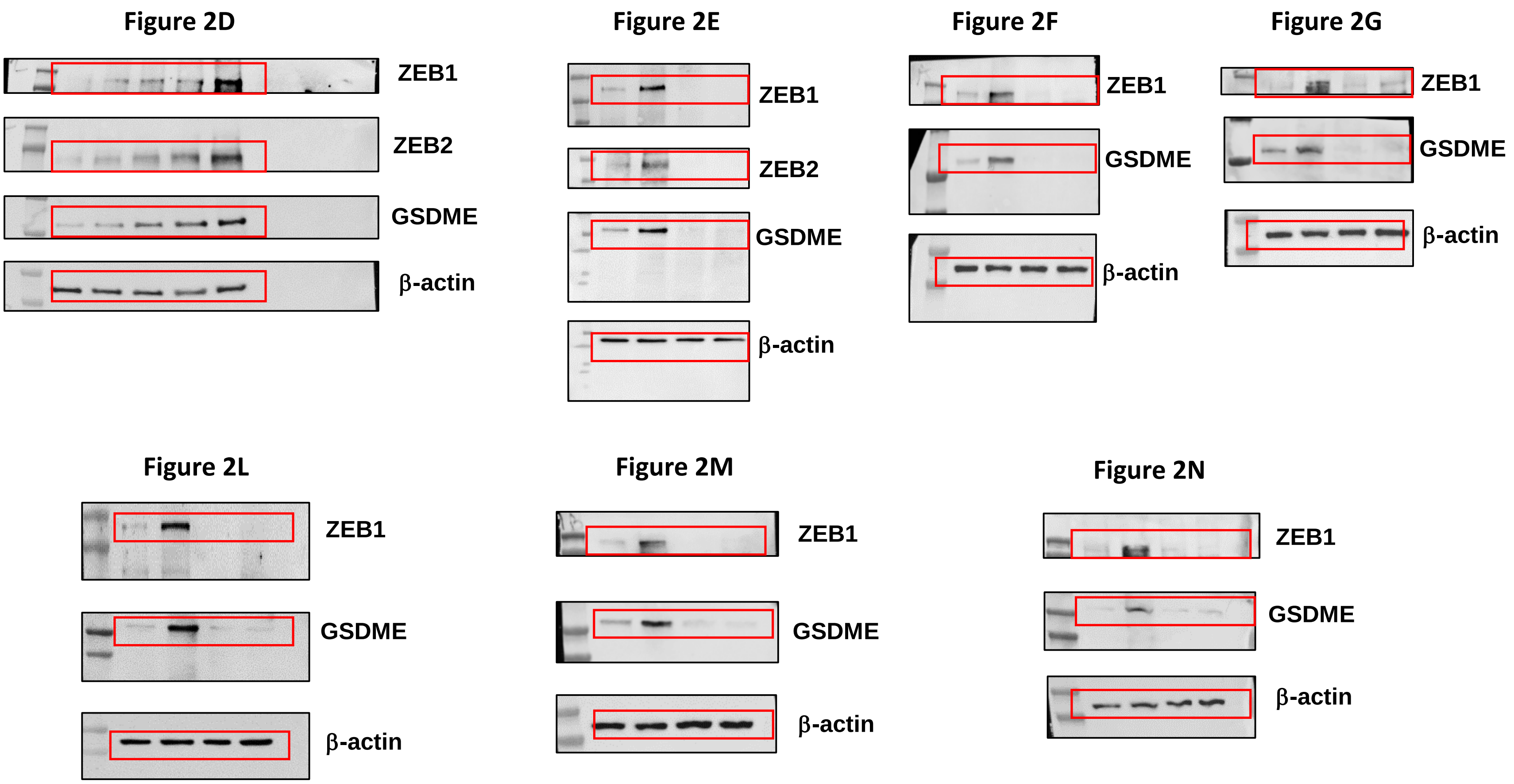
Figure 2D
ZEB1
ZEB2
GSDME
β-actin
Figure 2E
ZEB1
ZEB2
GSDME
β-actin
Figure 2F
ZEB1
GSDME
β-actin
Figure 2G
ZEB1
GSDME
β-actin
Figure 2L
ZEB1
GSDME
β-actin
Figure 2M
ZEB1
GSDME
β-actin
Figure 2N
ZEB1
GSDME
β-actin

Figure 3

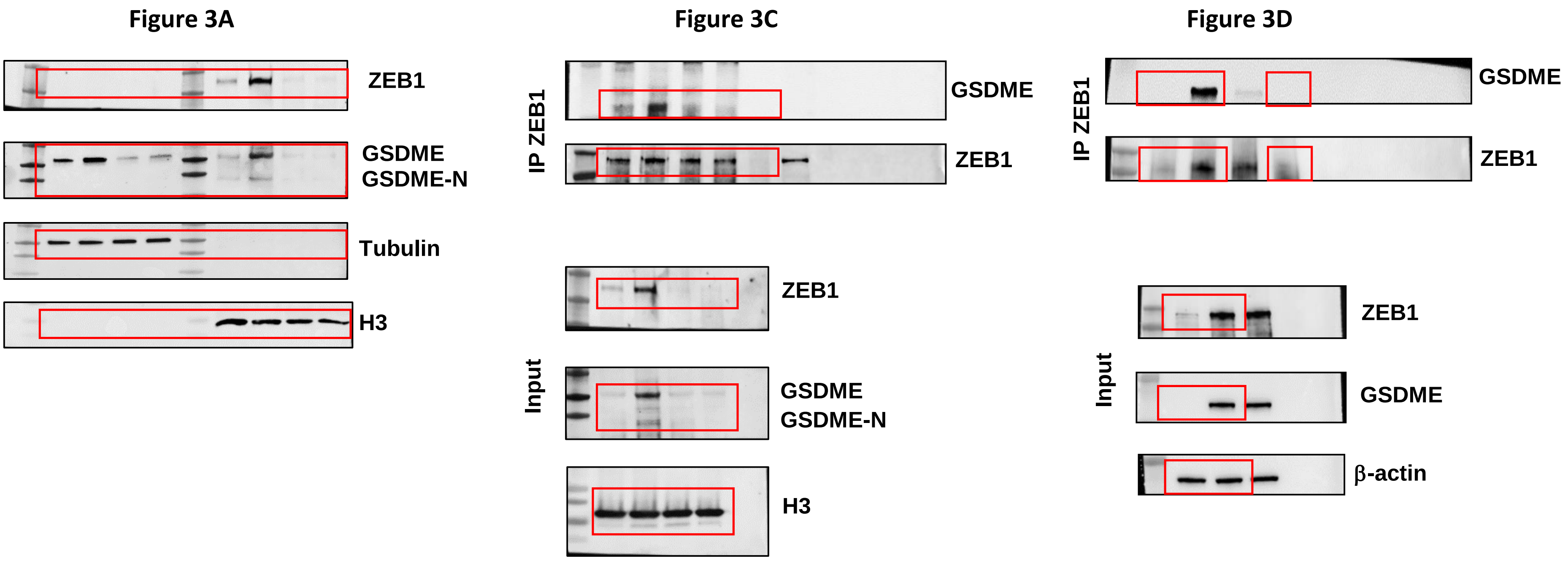

Figure 3A
ZEB1
GSDME
GSDME-N
Tubulin
H3
Figure 3C
IP ZEB1
GSDME
ZEB1
Input
ZEB1
GSDME
GSDME-N
H3
Figure 3D
IP ZEB1
GSDME
ZEB1
Input
ZEB1
GSDME
β-actin

Figure 4

Figure 4E

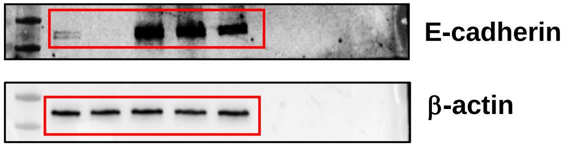

Figure 4H

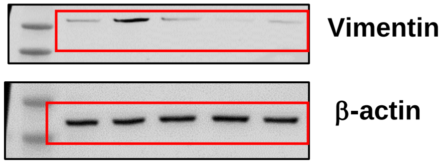

Figure 4K

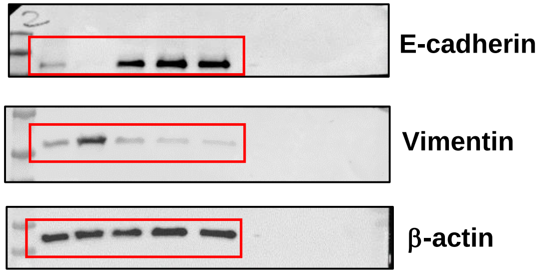

Figure 4L

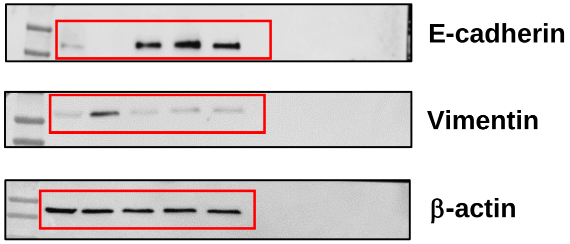

Figure 7

Figure 7C

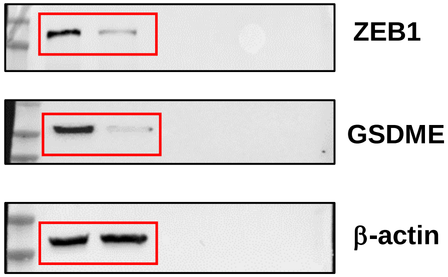

Figure 7F

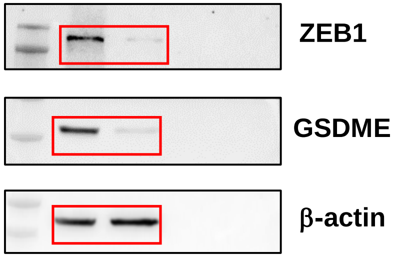

Supplementary Figure S1

Figure S1C

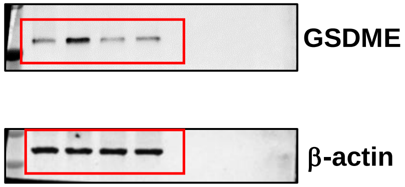

Supplementary Figure S2

Figure S2C

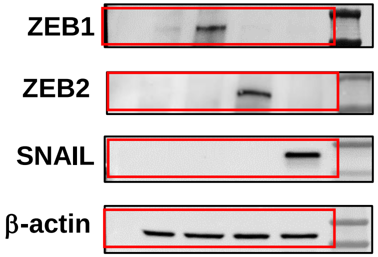

Figure S2D

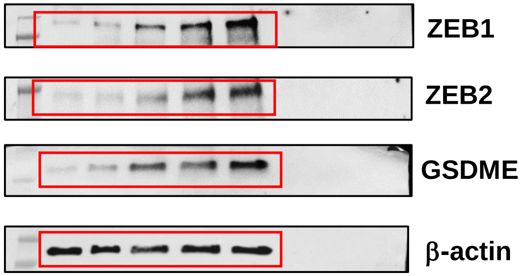

Figure S2E

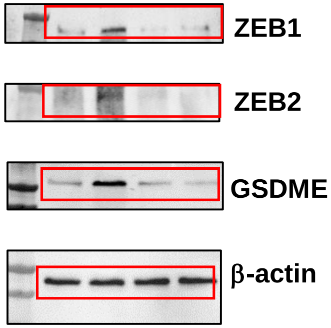

Figure S2F

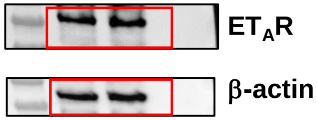

Figure S2G

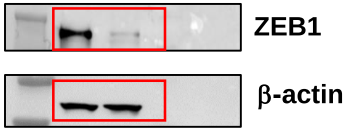

Figure S2H

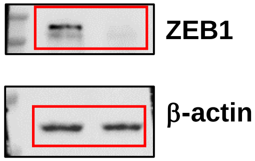

Figure S2J

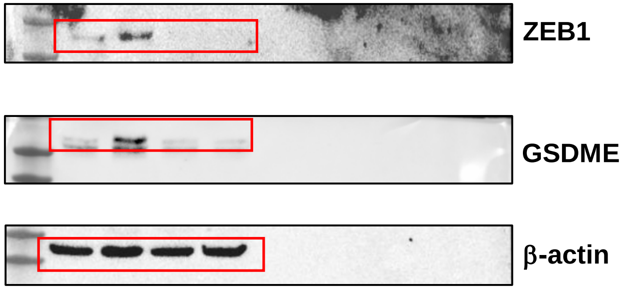

Supplementary Figure S3

Figure S3A

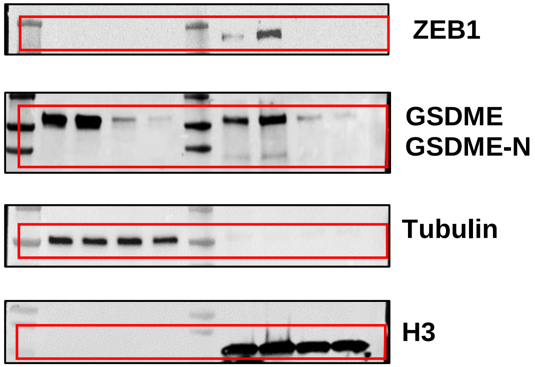

Figure S3B

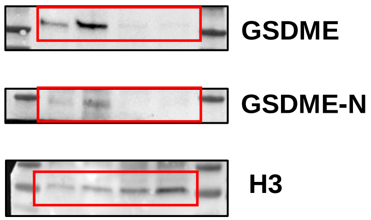

Figure S3C

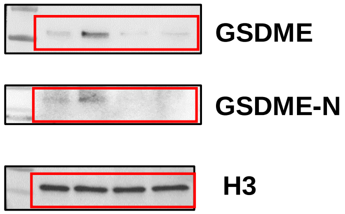

Figure S3D

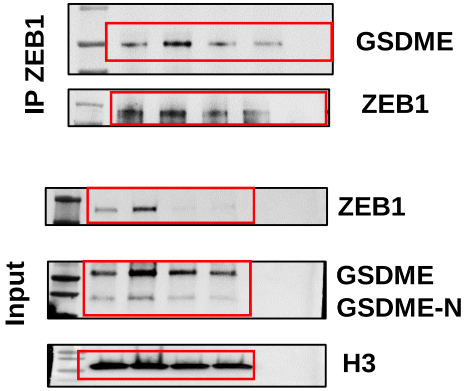

Supplementary Figure S4

Figure S4A

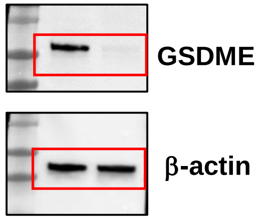

Figure S4B

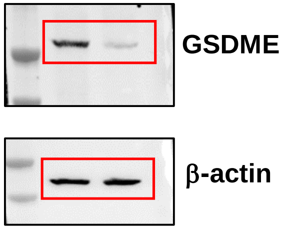

Figure S4C

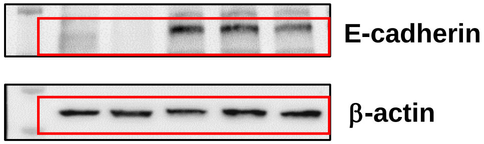

Figure S4E

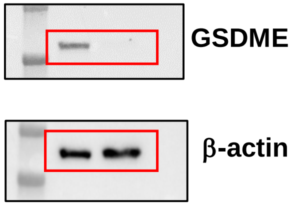

Figure S4F

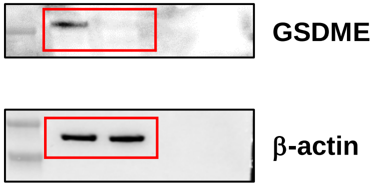

Supplement: Supplementary file 2 — Original western blots [file 41419_2025_8202_MOESM2_ESM.pdf]
